# Dataset of Near-infrared spectroscopy measurement for amylose determination using PLS algorithms

**DOI:** 10.1016/j.dib.2017.09.077

**Published:** 2017-10-06

**Authors:** P. Sampaio, A. Soares, A. Castanho, A.S. Almeida, J. Oliveira, C. Brites

**Affiliations:** a Instituto Nacional de Investigação Agrária e Veterinária (INIAV), Av. da República, Quinta do Marquês, 2780-157 Oeiras, Portugal; b Faculty of Engineering, Lusophone University of Humanities and Technology, Campo Grande, 376, 1749-019 Lisbon, Portugal

**Keywords:** Amylose, Chemometrics, Near-infrared, PLS, Rice

## Abstract

In the dataset presented in this article, 168 rice samples comprising sixteen rice varieties (including *Indica* and *Japonica* sub species) from a Portuguese Rice Breeding Program obtained from three different sites along four seasons, and 11 standard rice varieties from International Rice Research Institute were characterised. The amylose concentration was evaluated based on iodine method, and the near infrared (NIR) spectra were determined. To assess the advantage of Near infrared spectroscopy, different rice varieties and specific algorithms based on Matlab software such as Standard Normal Variate (SNV), Multiple Scatter Calibration (MSC) and Savitzky-Golay filter were used for NIR spectra pre-processing.

**Specifications Table**

**Table:** 

Subject area	Chemistry, Spectroscopy
More specific subject area	Amylose contents determination
Type of data	Data table,.mat file, figures
How data was acquired	Amylose concentration – spectrophotometry; Spectra - NIR transflection MPA equipment; Matlab software
Data format	Raw spectra data, analysed data, graphics
Experimental factors	168 rice samples and a pure sample of amylose were analysed. The amylose concentration was evaluated using the spectrophotometric technique, and the NIR spectra were obtained using an MPA – NIR transflection.
Experimental features	The amylose concentration was evaluated using the spectrophotometric technique. The Near infrared spectroscopy coupled with chemometric tools associated with Matlab software was used to treatment of data.
Data source location	Rice samples were harvested in Salvaterra de Magos, Alcácer do Sal and Montemor-o-Velho (Portugal).
Data accessibility	The data is available with this article.

**Value of the data**

• The data can be used as a supplement on the biochemical properties of amylose concentration and can be compared with other related studies.
• Those data establish a link between biochemical properties and reflectance spectra on several rice samples for amylose evaluation using different PLS model.
• Several Matlab algorithms such as SNV, MSC, derivatives and others Savitzky-Golay filters allowed to preprocessed the raw NIR spectra.
• The experimental data of amylose and NIR spectra can be used for analysis of different PLS algorithms (iPLS, siPLS and mw-PLS).

## 1 Data

**Fig. 1:**
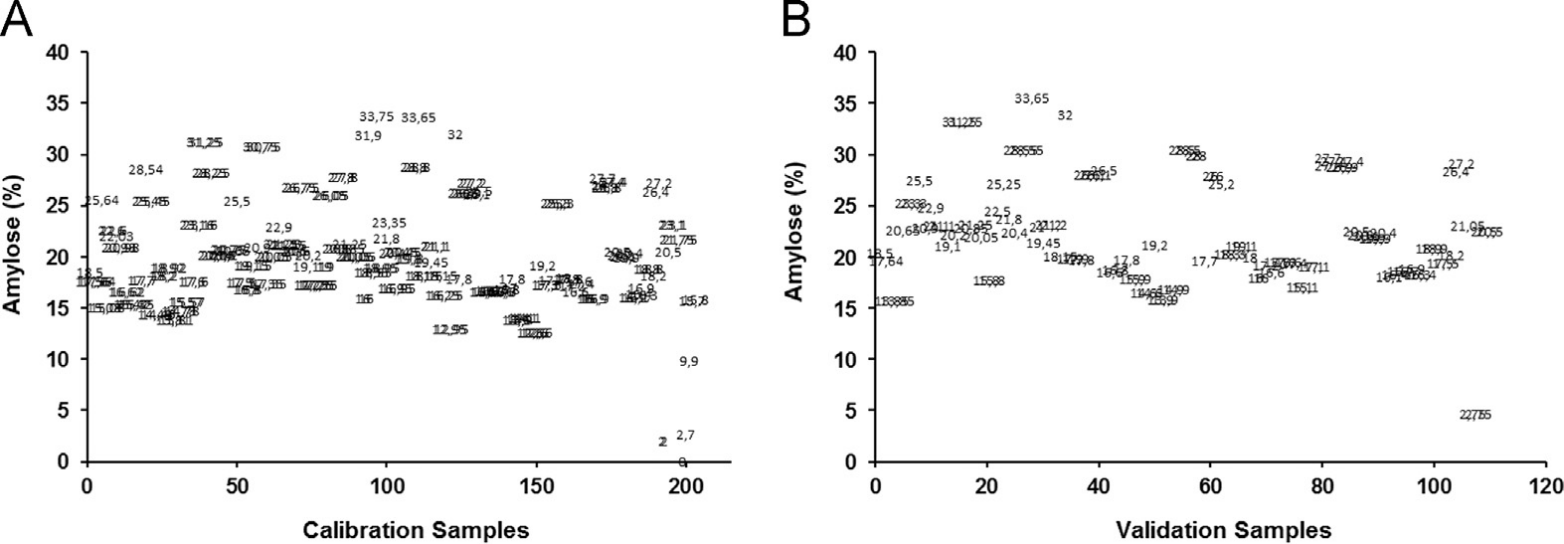
Graphical representation of rice samples used for calibration step (A) and validation step (B) related to amylose model.

Amylose concentration of 168 different rice samples was determined using a spectrophotometric method (Fig. 1A and B). For the same samples the NIR spectra were obtained using the Spectra – NIR transflection MPA equipment (Fig. 3A–B, Matlab file: RawData.mat). After that, the spectra data were previously analysed by principal component analysis method for identifying and removing the outliers and consequently the samples were divided in calibration (Figs. 1A and 3B) and validation data (Figs. 1B and 3B). The data file was evaluated using the Matlab toolbox for spectra pretreatments such as Standard Normal Variate transformation (SNV), Multiple Scatter Calibration (MSC) and Savitzky-Golay filters.

## 2 Experimental design, materials and methods

### 2.1 Rice sample

For this study, 20 g of rice was grounded to flour in a Cyclone Sample Mill (Falling number 3100, Perten, Sweden) equipped with a 0.8 mm screen. Sixteen rice varieties (including *Indica* and *Japonica* sub species) from a Portuguese Rice Breeding Program were grown in three very different micro climates (Alcácer do Sal, Salvaterra-de-Magos and Montemor-o-Velho, Portugal) along four seasons (2012–2015), providing 168 samples. For each rice sample, the amylose was determined in duplicate. In addition, 11 standard rice varieties, sourced from the International Rice Research Institute, Los Baños, Philippines, (IRRI), characterized by different amylose content, were also used: IR65; IR24; IR64; WU BAI LI; IRRI109; IRRI134; IRRI138; IRRI148; IRRI149 and IRRI151. The samples used for the calibration step are represented in the Fig. 1A, while the samples used for validation process are represented in the Fig. 1B.

### 2.2 Amylose determination

**Fig. 2:**
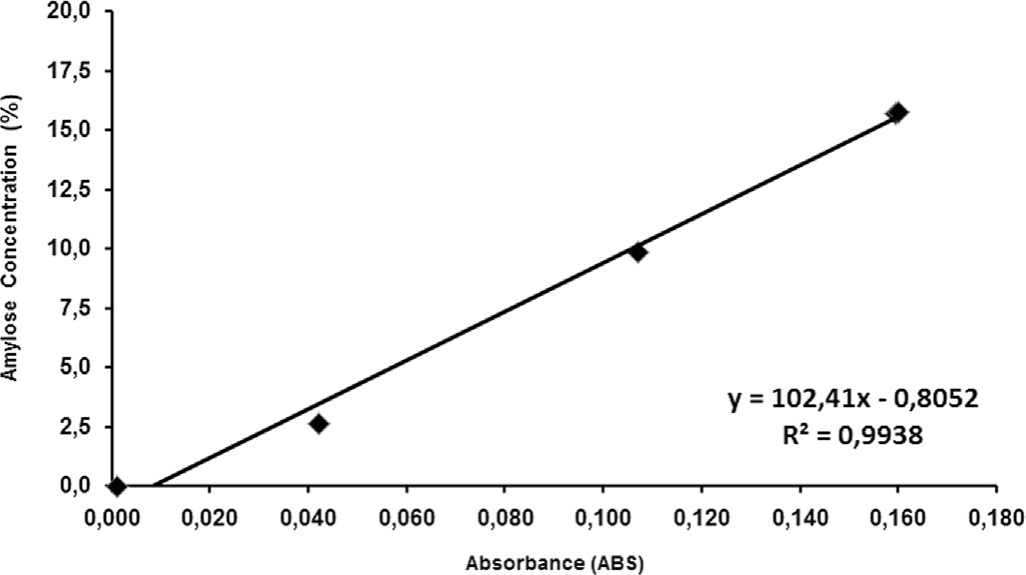
Calibration plot for amylose concentration evaluation.

The amylose concentration was determined using the standard iodine colorimetric method prepared according to ISO 6647-2 [1]. The absorbance was measured using a spectrophotometer (Hitachi, Japan) at 720 nm. Amylose content was quantified using a standard curve created from absorbance values of 4 calibrated samples from standard rice varieties (IR65, IR24, IR64, IR8) obtained from IRRI (Fig. 2). The calibration values were obtained by separation of hydrodynamic volume and molecular weight of amylose by size exclusion chromatography ISO 6647-1 [2].

### 2.3 Instrumentation and measurements

**Fig. 3:**
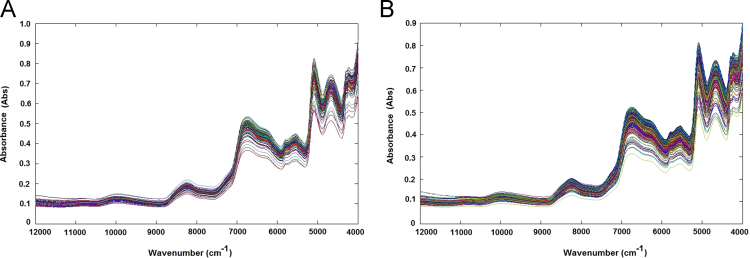
NIR spectra without any preprocessing step related to samples of validation step (A) and samples used for calibration step (B).

The samples containing approximately 25 cm^3^ of rice flour were loaded in a circular sample cup and pressed slightly to obtain a similar packing density. Sample spectra were registered using an NIR transflection MPA equipment (Bruker Optics, Germany). For each rice sample, 16 successive scans were performed, over a wavenumber range (12,000–4000 cm^−1^), at 16 cm^−1^ of resolution. For each rice sample two spectra were obtained (Fig. 3A and B).

### 2.4 Principal component analysis (PCA)

**Fig. 4:**
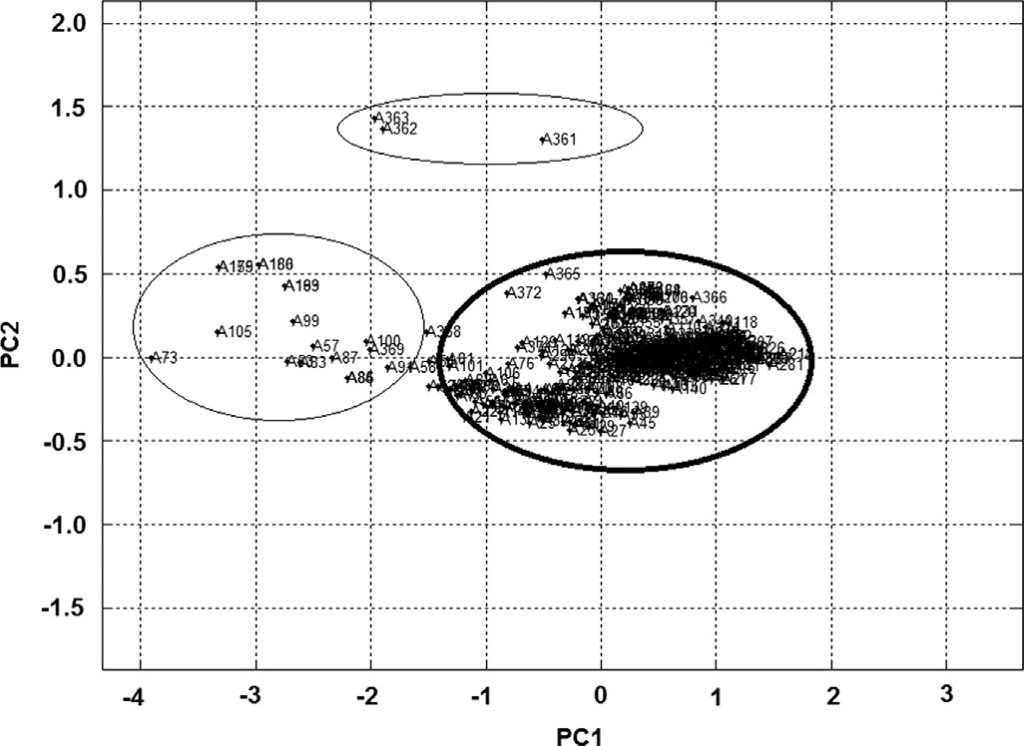
PCA analysis of total samples allowing to identify the outliers samples.

Principal component analysis is a linear pattern recognition technique that allows the reduction of the dimensionality of multivariate data to *n* principal components. All samples were considered for analysis to enable inferring how sample variability may affect possible trends from the direct observation of the scores plot. The outliers were identified using PCA analysis. PCA was performed using MATLAB® 7.9.0 software (Matlab-toolbox). PCA analysis was performed to select the suitable experimental data for model construction and to identify and eliminate the outliers (Fig. 4).

### 2.5 Data and preprocessing algorithms

**Fig. 5:**
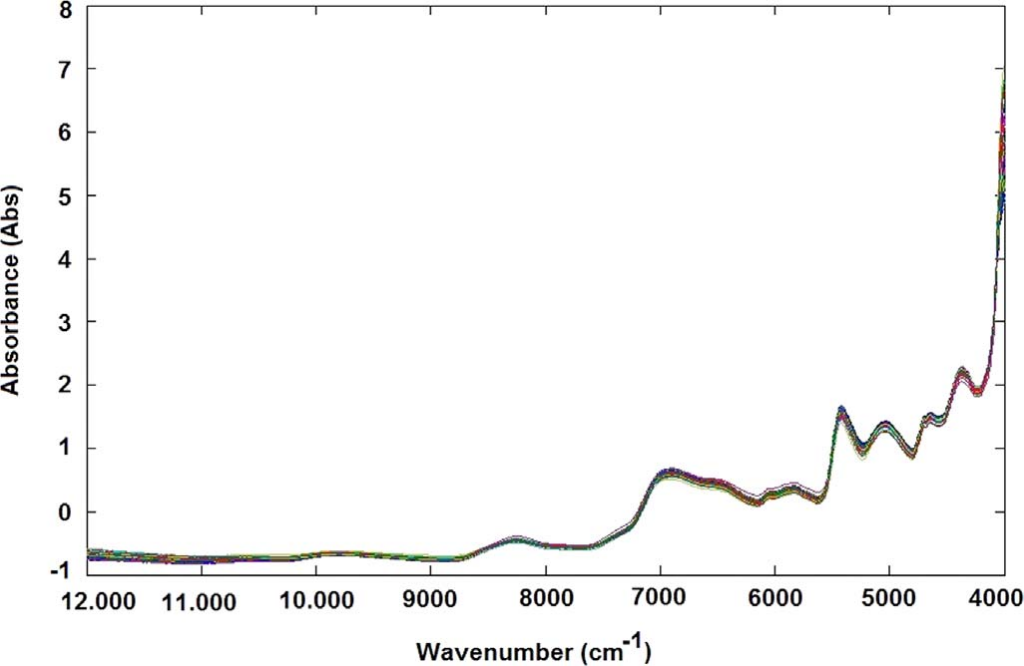
NIR spectra pre-processed using the SNV method.

**Fig. 6:**
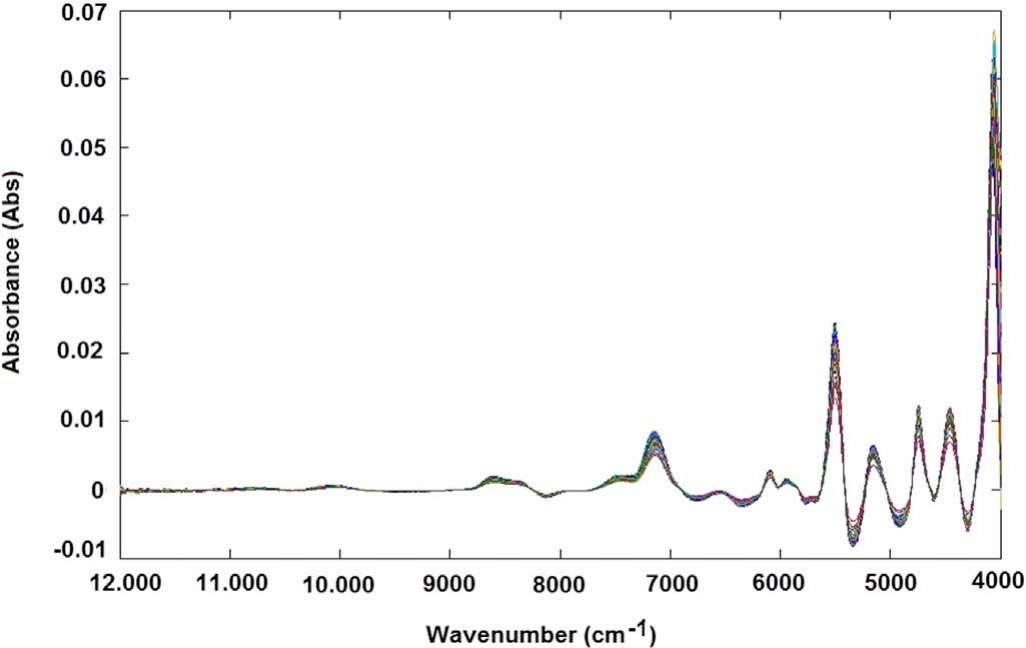
NIR spectra pre-processed using the first-derivative method.

**Fig. 7:**
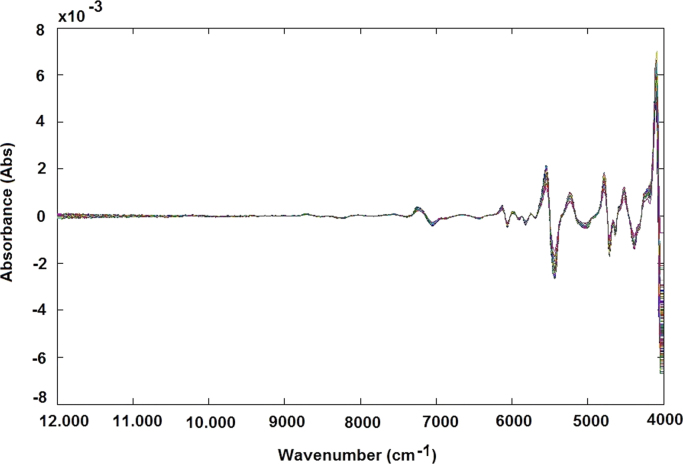
NIR spectra pre-processed using the second-derivative method.

**Fig. 8:**
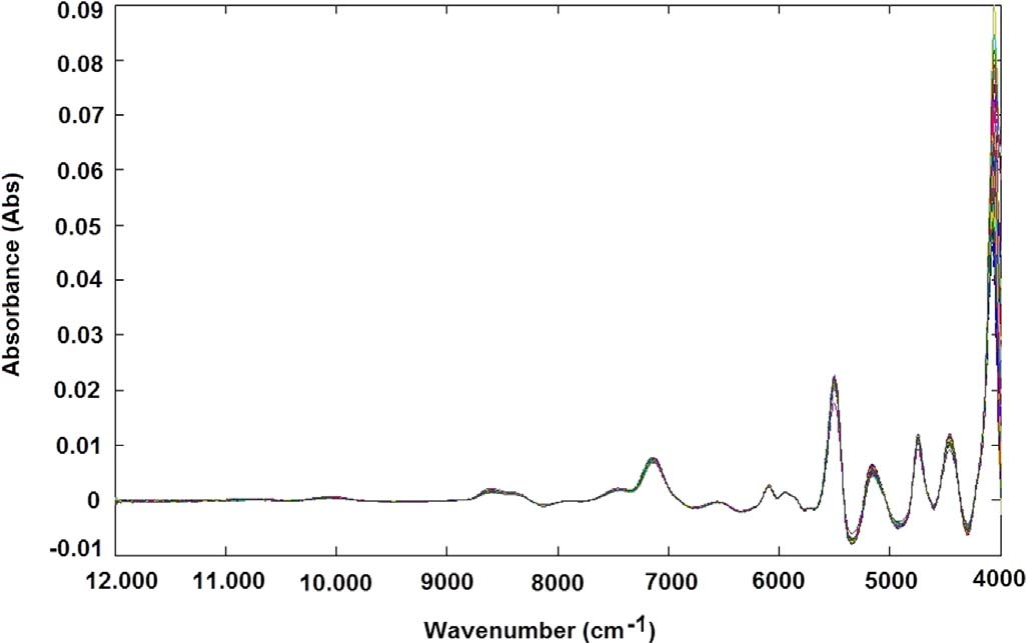
NIR spectra pre-processed using the MSC plus first-derivative method.

**Fig. 9:**
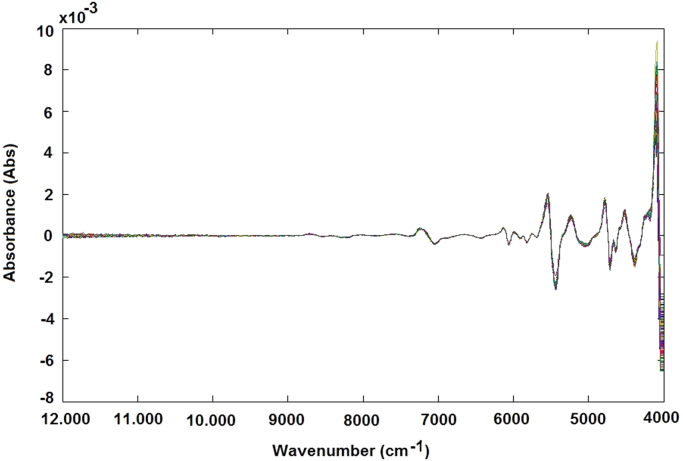
NIR spectra pre-processed using the MSC plus second-derivative method.

**Fig. 10:**
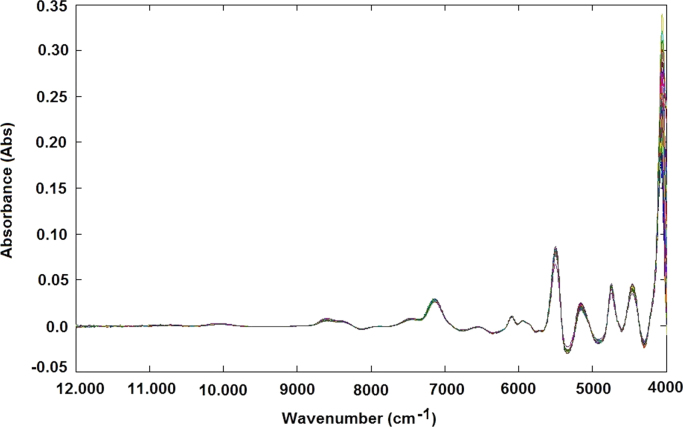
NIR spectra pre-processed using the SNV plus first-derivative method.

**Fig. 11:**
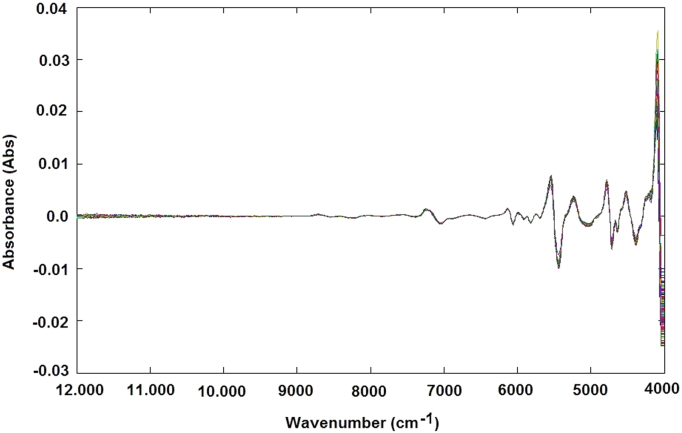
NIR spectra pre-processed using the SNV plus second-derivative method.

**Fig. 12:**
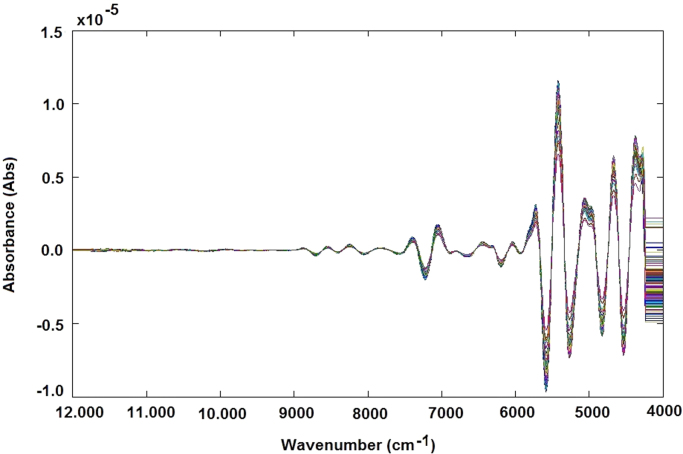
NIR spectra pre-processed using the Savitzky-Golay filter.

**Fig. 13:**
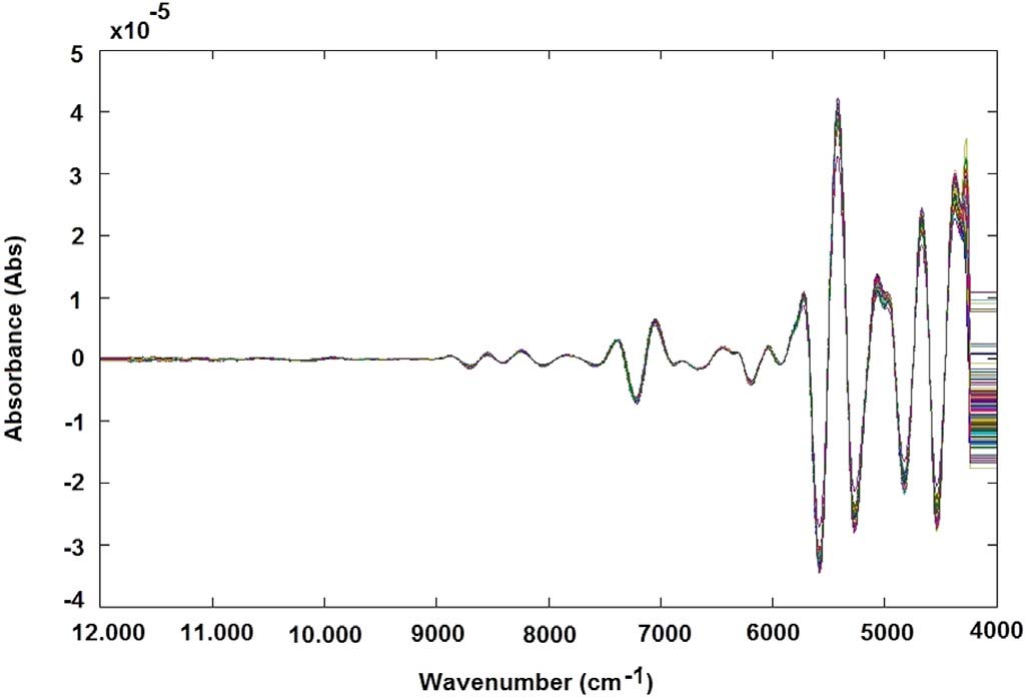
NIR spectra pre-processed using the SNV plus Savitzky-Golay filter.

The NIR raw spectra obtained, after outlier exclusion, were treated by different data preprocessing techniques, such as standard normal variate (SNV) transformation (Fig. 5), and smoothing derivative (first derivative) (Fig. 6) and (second derivative) (Fig. 7) to obtain reliable qualitative classification and quantitative calibration models. After the MSC and SNV, the spectra were also treated using first and second derivatives: MSC plus 1st derivative (Fig. 8) MSC plus 2nd derivative (Fig. 9), and SNV plus 1st derivative (Fig. 10) and SNV plus 2nd derivative (Fig. 11). Savitzky-Golay smoothing method allowed eliminate the noises like baseline-drift, tilt, reverse, and so forth [3], [4] (Fig. 12). Consequently, the Savitzky-Golay filter was applied after SNV treatment, respectively (Fig. 13) (Matlab-toolbox).

## 3 Amylose determined using the siPLS model

The rice samples were evaluated regarding the NIR spectroscopy, and the spectra were used for building the siPLS model for amylose prediction in rice. The model created PLS is particularly useful to predict a set of dependent variables from a (very) large set of independent variables (i.e., predictors). Due to the large number of rice samples, the experimental data related to colorimetric method of all samples used in this study, as well as the correspondent value obtained through the siPLS model developed from the NIR spectra were submitted as in the Excel file (DatainBrief_AmyloseContents).
