# Supplementary material for: Dataset of Near-infrared spectroscopy measurement for amylose determination using PLS algorithms
Source: Data Brief. 2017 Oct 6;15:389–96. doi: 10.1016/j.dib.2017.09.077 (PMC5712058; doi:10.1016/j.dib.2017.09.077)
Supplement: Supplementary file 1 — Transparency document [file mmc1.docx]

**Conflict of the Interest Form**

**Statement**

**Ms. Ref. No.:**  DIB-D-17-0884

**Title:** Dataset of Near-infrared spectroscopy measurement for amylose determination using PLS algorithms

The authors sate that there is not any conflict of the interest about this experimental work.

Best regards,

Pedro Sousa Sampaio
