# Supplementary material for: Dataset of Near-infrared spectroscopy measurement for amylose determination using PLS algorithms
Source: Data Brief. 2017 Oct 6;15:389–96. doi: 10.1016/j.dib.2017.09.077 (PMC5712058; doi:10.1016/j.dib.2017.09.077)
Supplement: Supplementary file 4 — Supplementary material [file mmc4.zip › TOOLBOX/iToolbox_Manual2013.pdf]

iToolbox Manual

by

Lars Nørgaard
Email: lno@foss.dk
Web: www.models.life.ku.dk

March 2013

Contents

Important notes on the iToolbox for MATLAB....3
iToolbox – Getting Started....4
A word on over fit and outliers....4
Help....4
Interval PLS – how to do it....5
Backward interval PLS....10
biPLS as preprocessing for genetic algorithms....10
Moving window PLS....10
Synergy interval PLS....11
Interval PCA....11
PLS models on selected intervals and prediction....12
Validation....13
Preprocessing....13
Model structure....14
m-files alphabetically....15
iToolbox directory....15
Auxiliary files....17

Important notes on the iToolbox for MATLAB

Conditions

The toolbox is freeware. References to the implemented methods are given below.

Authors

Lars Nørgaard, lno@foss.dk, FOSS
Affiliated professor in Chemometrics Group, Quality & Technology
University of Copenhagen
Denmark

&

Riccardo Leardi, riclea@dictfa.unige.it (bipls & bipls_dyn)
Department of Pharmaceutical and Food Chemistry and Technology
University of Genoa
Italy

References

L. Nørgaard, A. Saudland, J. Wagner, J.P. Nielsen, L. Munck and S.B. Engelsen, Interval Partial Least Squares Regression (iPLS): A Comparative Chemometric Study with an Example from Near-Infrared Spectroscopy, Applied Spectroscopy, 54, 413-419, 2000.

R. Leardi and L. Nørgaard, Sequential application of backward interval-PLS and Genetic Algorithms for the selection of relevant spectral regions, Journal of Chemometrics, 18, 486-497, 2004.

Warranty

In short, no guarantees, whatsoever, are given for the quality of this toolbox or for the consequences of its use.

Setting up the toolbox

In order to install the toolbox just copy all the files to a directory (e.g. iToolbox) and add this to your MATLAB path.

Support

We are very interested in and dependent on feedback from the users (preferably by e-mail). If you have problems running the toolbox please supply screen dumps as well as version number of the toolbox, MATLAB, and operating system before contacting us. We will do the utmost to help overcoming the problems.

iToolbox – Getting Started

The iToolbox is for exploratory investigations of data sets with many collinear variables (e.g. spectral data sets). The main methods in the iToolbox are

interval PLS (iPLS): Splits the data set into a number of intervals (variable-wise), calculates PLS models for each interval and presents the results in one plot. This method is intended to give an overview of the data and can be helpful in interpretation (e.g. for spectral assignments).

backward interval PLS (biPLS): As in the interval PLS model the data set is split into a given number of intervals, but now PLS models are calculated with each interval left out, i.e. if one chooses 20 intervals then each model is based on 19 intervals leaving out one interval at a time. The first left out interval is the one that when left out gives the best performing model with respect to RMSECV or RMSEP (Root Mean Square Error of Cross Validation / Prediction). This procedure is continued until one interval remains. The results are presented in a table.

moving window PLS (mwPLS): Calculates iPLS models based on a moving window concept. For each variable a PLS model is calculated with the given window size. The results are presented in a plot.

synergy interval PLS (siPLS): Splits the data set into a number of intervals (variable-wise) and calculates all possible PLS model combinations of two, three or four intervals. The computation time can be long depending on the number of intervals and the selected number of intervals to combine. The results are presented in a table.

interval PCA (iPCA): Splits the data set into a number of intervals (variable-wise), calculates PCA models for each interval and presents the results in multiple score plots. This method is intended to give an overview of the data and can be helpful in exploratory studies and interpretation (e.g. when looking for groupings among samples).

PLS models and prediction: To be used for developing PLS models on selected intervals from iPLS and biPLS and for prediction based on new data sets.

Try out the demos to get an impression of how to use the methods: **iplsdemo**, **biplsdemo**, **mwplsdemo**, **siplsdemo**, **ipcademo**.

A word on over fit and outliers

Please be aware that the methods implemented in this toolbox might over fit the data. To be completely safe, e.g. when using the models for predictive purposes, an independent test set should always be evaluated to see if the results obtained correspond to the results found by using the toolbox. This is not a special case for this toolbox but goes for all variable selection methods.

A large number of PLS models are calculated in each of the methods and it is important to be aware that outliers can distort the results as they can in standard PLS modeling.

Help

In general you can write the name of the file to get help on its function. E.g. if you write **ipls** at the MATLAB prompt followed by [ENTER] you get the following text:

```
Model=ipls(X,Y,no_of_lv,prepro_method,intervals,xaxislabels,
val_method,segments);
```

```
Example:

Model=ipls(X,Y,7,'mean',20,xaxis,'syst123',5);
```

If you write **help ipls** you get extended help, including input-output (I/O) explanation, and this goes for all relevant files.

If the name of directory where the toolbox files are placed is, e.g., iToolbox, and this has been added to the MATLAB path (**File**, **Set Path** in MATLAB) you can write **help iToolbox** to get a list of all relevant files.

Interval PLS – how to do it

The data used for illustration are real extract measurements on beer samples (ycal) as well as corresponding near infrared (NIR) spectroscopic data (Xcal) in the range 400-2250 nm (every 2nd nm is recorded). In the calibration set the number of samples is 40. An independent test set is available consisting of 20 samples (Xtest & ytest).

Load the data:

```
load nirbeer
```

Make a plot of the raw data:

```
plot(xaxis,Xcal)
```

The plot should look like this:

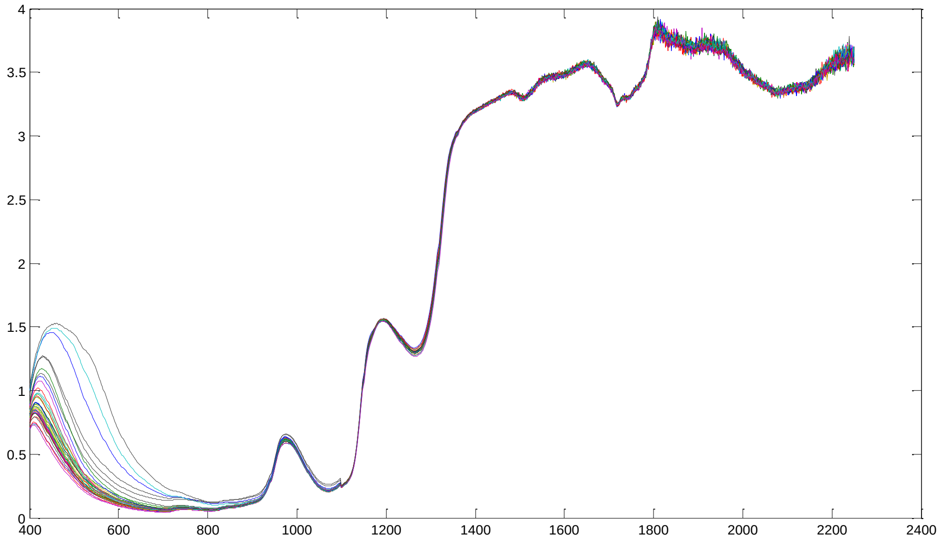

The experienced spectroscopist would immediately see the noisy part starting at around 1500 nm, and would probably exclude this part before analysis. In this case we keep it and will show how iPLS brings us to the same conclusion as the spectroscopist.

Make an iPLS model with e.g. 20 intervals using **ipls**:

```
Model=ipls(Xcal,ycal,10,'mean',20,xaxis,'syst123',5);
```

By this command 20 models are calculated, all of them are cross validated with five segments and systematic exclusion (1,6,11 etc.). The models are mean centered and up to 10 PLS components (PLSC) are calculated. The xaxis vector contains the wavelengths and should have the same number of variables as Xcal.

The number of PLSC to use in a global model can be estimated by using **plsrmse** and **plspvsm** with the parameter 0:

```
plsrmse(Model,0)
```

This yields the following plot:

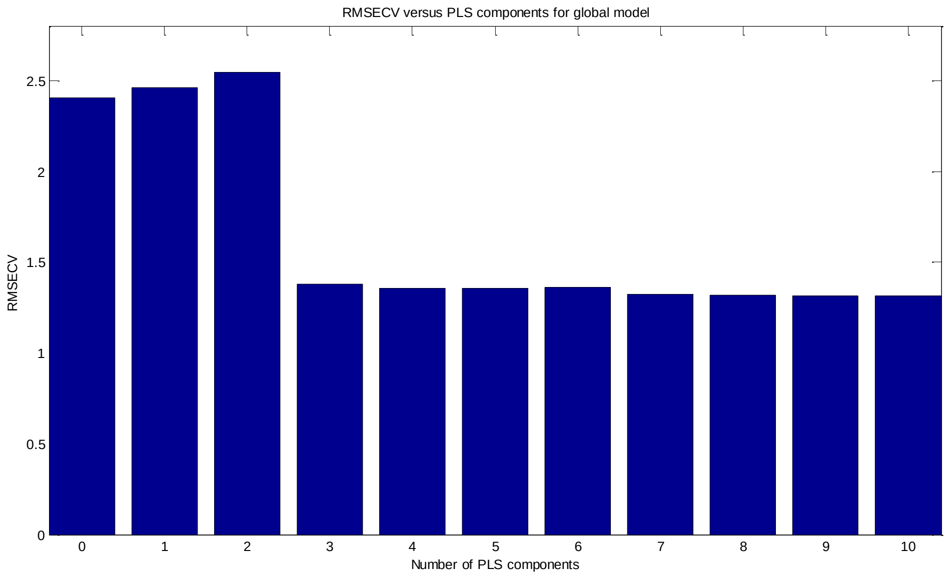

In this plot a minimum is seen around four PLSC but we also notice problems since the RMSECV for one and two PLSC is larger than that obtained at zero PLSC. This might be due to the noisy part of the spectrum or outliers.

Let's make the iPLS plot with four PLSC for the global model:

```
iplsplot(Model,'intlabel',4)
```

The plot looks like this:

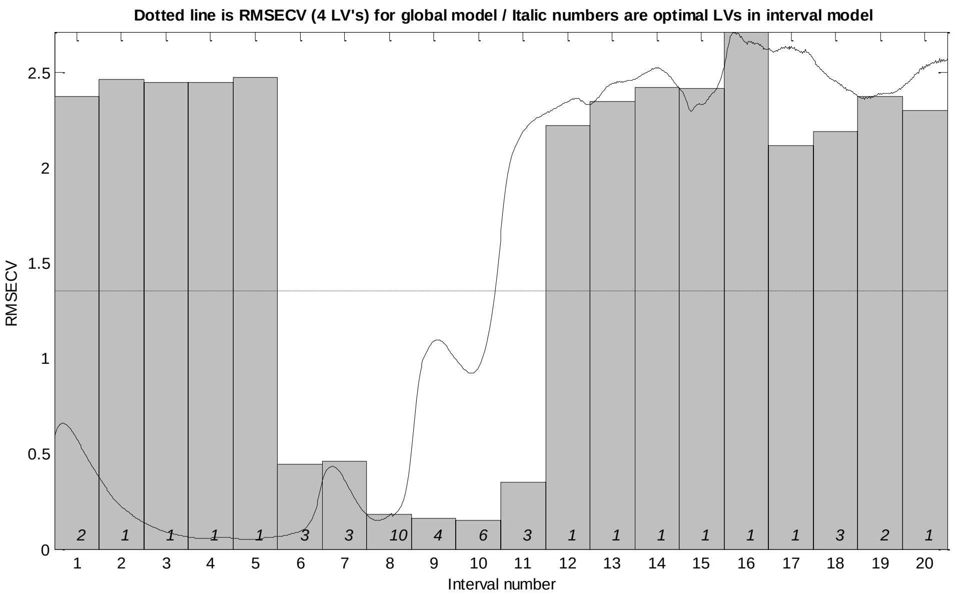

The italic number on each bar indicates the optimal number of PLSC in each interval and the dotted line is the RMSECV of the global model with four PLSC.

As seen from the plot intervals 6-11 performs better than the global model. The visual part of the spectrum which is very systematic doesn't contain information about the real extract.

If we prefer to see the wavelengths instead we can make the plot as follows:

```
iplsplot(Model,'wavlabel',4)
```

And the plot looks like this:

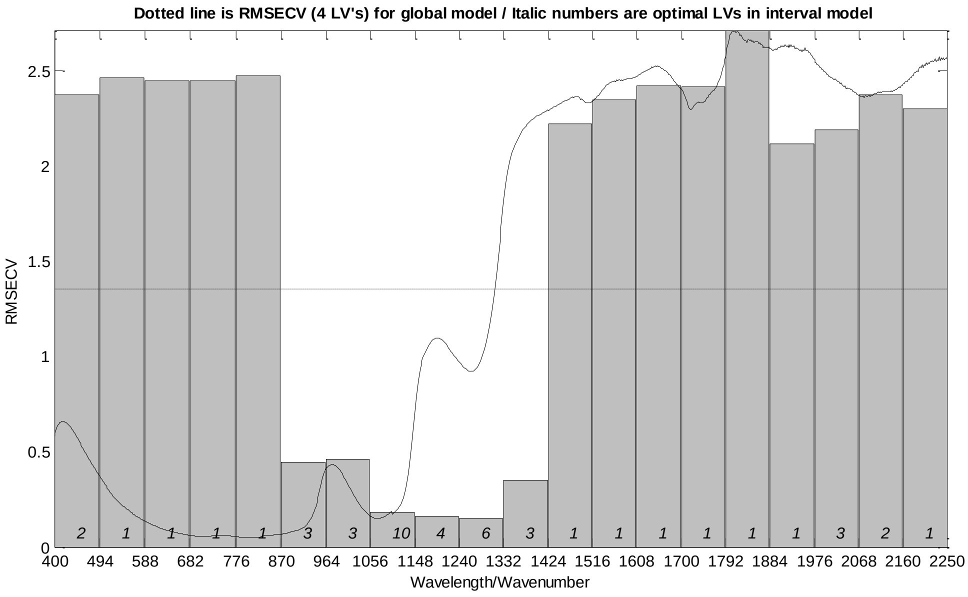

If you want to investigate a single interval in more details use **plsrmse** for that interval as well as **plspvsm**. If we want to have a closer look at interval 10 with 6 PLSC we write:

```
plspvsm(Model,6,10)
```

Two plots are produced:

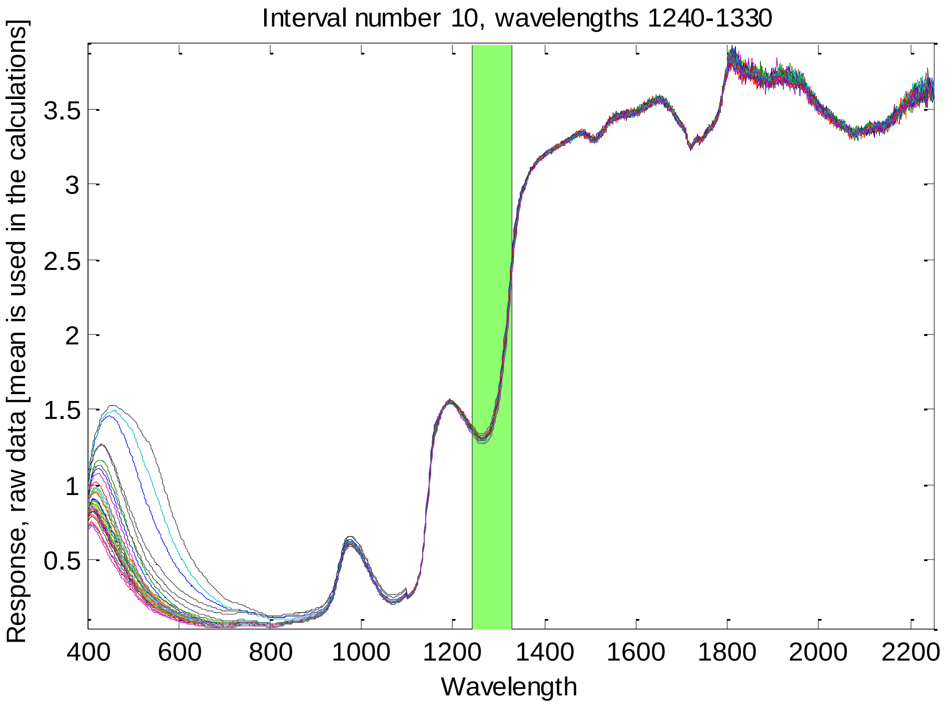

and

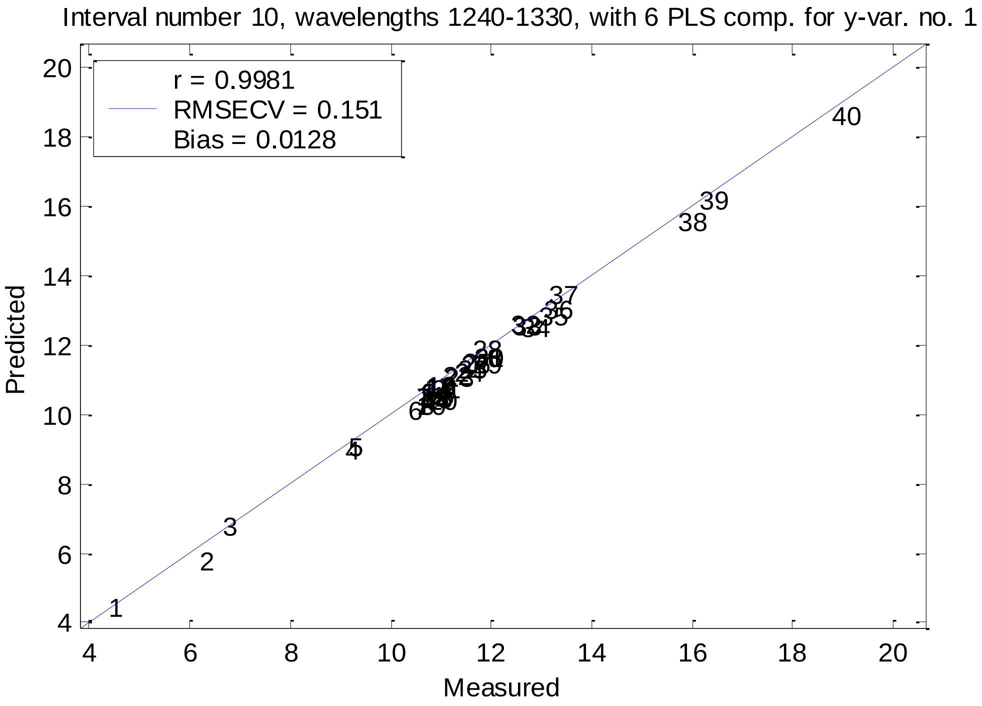

To display exact information about intervals (variable numbers, wavelengths etc.) use **intervals(Model)**:

Interval	Start var.	End var.	Start wav.	End wav.	Number of vars.
1	1	47	400	492	47
2	48	94	494	586	47
3	95	141	588	680	47
4	142	188	682	774	47
5	189	235	776	868	47
6	236	282	870	962	47
7	283	328	964	1054	46
8	329	374	1056	1146	46
9	375	420	1148	1238	46
10	421	466	1240	1330	46
11	467	512	1332	1422	46
12	513	558	1424	1514	46
13	559	604	1516	1606	46
14	605	650	1608	1698	46
15	651	696	1700	1790	46
16	697	742	1792	1882	46
17	743	788	1884	1974	46
18	789	834	1976	2066	46
19	835	880	2068	2158	46
20	881	926	2160	2250	46
21	1	926	400	2250	926

Note that you can specify manually your intervals; see **makeManualIntervals** script-file for an example. In the present example we might want to investigate the visual part, the NIR part without noise and the NIR part with noise; i.e. three intervals. By using **int_vec=[1 200 201 500 501 926]** as input to the ipls file like this:

```
Model=ipls(Xcal,ycal,10,'mean',int_vec,xaxis,'syst123',5);
```

We get the following iplsplot by typing

```
iplsplot(Model,'wavlabel',4)
```

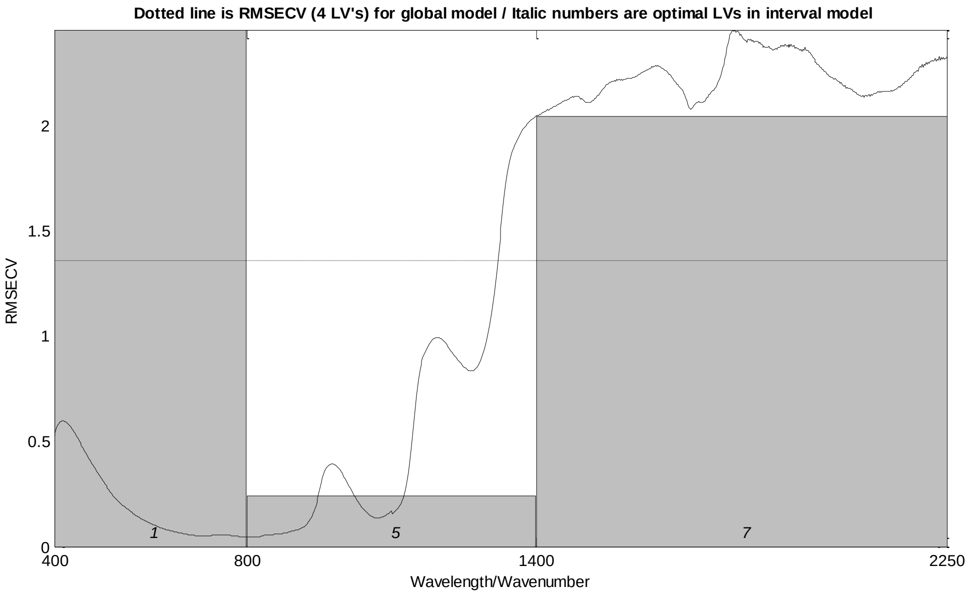

Note that the abscissa labels have to either `'varlabel'` or `'wavlabel'` the intervals are not shown correct with `'intlabel'`.

By **makeManualSegments** you can define exactly how you would like to do segmented cross validation (this is a script-file so you have to edit it to suit your data before input to **ipls**).

If you want to perform a test validation in stead of cross validation just give the test set samples index as an input and change `'syst123'` to `'test'`:

```
Model=ipls(Xcal,ycal,10,'mean',20,xaxis,'test',(2:2:38)');
```

The statement `(2:2:38)'` gives the index of the test set samples as a column vector.

Backward interval PLS

Make a backward iPLS model with a given number of intervals using **bipls**:

```
biModel=bipls(Xcal,ycal,10,'mean',20,[],'syst123',5);
```

Then use **biplstable(biModel)** to see a table of RMSECVs/RMSEPs.

biPLS as preprocessing for genetic algorithms

Use **bipls_dyn** to get an overview of the relevant variables. Based on a plot of the output vector from **bipls_dyn** you can decide the variables to include in the GA. The output vector contains for each variable the number of times the variable is selected in the dynamic backward interval PLS modelling.

Moving window PLS

Make a moving window PLS model with a given windowsize using **mwpls**:

```
mwModel=mwpls(Xcal,ycal,7,'mean',31,[],'syst123',5);
```

Then use **mwplsplot** to plot the RMSECVs/RMSEPs for each variable and **mwpls_comp** to plot number of component used in the optimal models.

```
mwplsplot(mwModel)
```

gives the following plot

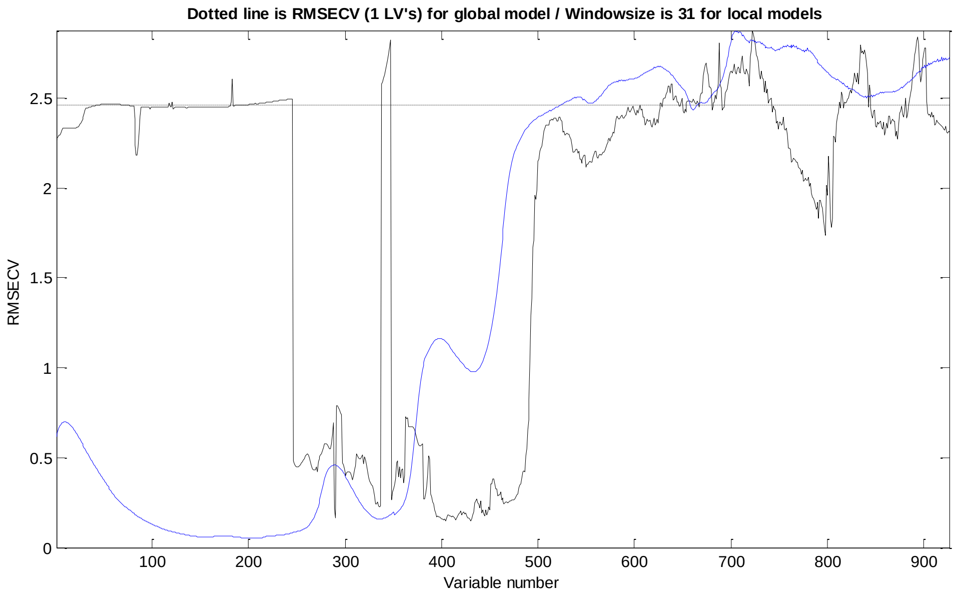

The black line is the RMSECVs/RMSEPs for each model based on a window of 31 variables.

Synergy interval PLS

Use **sipls** to calculate all PLS models on all combinations of 2, 3 or 4 intervals. List the results by **siplstable**. Next, (re)make PLS models on selected interval combinations with **plsmodel** (you can use this function for any combination of intervals, i.e. also more than four). Study the results by **plsrmse** and **plspvsm**.

Interval PCA

Make an interval PCA model using **ipca**:

```
Model=ipca(Xcal,7,'mean',20,xaxis);
```

Use **makeClasses** to produce an index vector for groups in the data. In this case we are interested in seeing if samples with extract values below 11 can be discriminated from samples with values above 11. A vector designated `classes` is produced for this purpose. Use this as input to **ipcascoplot**

```
ipcascoplot(Model,1,2,[],classes)
```

Now five figures are produced with PC1 versus PC2 plots for 20 intervals (Figure 2-5) as well as the global model (Figure 1). By going through the plots it is observed that interval 11 seems optimal for discriminating the two groups in PC1 versus PC2. If you want to see other PC combinations just change the input to **ipcascoplot**.

If you want only one plot pr. figure simply write:

```
ipcascoplot(Model,1,2,[],classes,1)
```

and 21 figures are produced.

Use **ipcascoplotall** to plot score plots for all possible combinations up to the given number of principal components.

To see the corresponding loadings use **ipcaloadplot**.

PLS models on selected intervals and prediction

If one wants to investigate a model based on e.g. intervals 9 and 10 as judged from an interval PLS model **plsmodel** can be used:

```
oneModel=plsmodel(Model,[9 10],10,'mean','syst123',5);
```

where `Model` is the output from the ipls calculation (or the bipls calculation). The results can be evaluated by **plsrmse** and **plspvsm** just as for the iPLS model. Try

```
plspvsm(oneModel,3)
```

to see the results from a three PLSC model on intervals 9 and 10. This model can be used for prediction of an independent test:

```
predModel=plspredict(Xtest,oneModel,3,ytest);
```

Also this model can be evaluated by **plsrmse** and **plspvsm** if `ytest` is known. If you want to see the actual predictions for e.g. three PLSC write

```
predModel.Ypred(:,:,3)

ans =

    3.9564
```

```
 7.1407
10.2851
10.3603
10.4527
10.2982
10.5956
10.6302
10.6746
10.4699
11.0596
11.2307
11.6149
11.2665
11.4898
11.9501
12.4822
12.9380
15.7439
16.0137
```

Validation

Regression models are validated by either cross validation or dependent test set validation. Cross validation can be performed as standard full CV, systematic 123 (Venetian blinds), systematic 111 (contiguous blocks), random or manual. For the manual CV please consult the makeManualSegments script file to see how manual segments are specified. For test set validation the test set samples should be given by an index column vector. E.g. (2:2:40)' uses every 2nd sample in the test set.

When models are developed by any of the regression methods it is recommended to test the models with an *independent* test set. Use plsmodel and plspredict for this.

Preprocessing

The preprocessing methods implemented are none (`'none'`), mean centering (`'mean'`), autoscaling (`'auto'`), multiplicative signal correction (MSC) + mean centering (`'mscmean'`), and MSC + autoscaling (`'mscauto'`).

Note that MSC is performed followed by either mean centering (`'mscmean'`) or auto scaling (`'mscauto'`). When used with **ipls**, **mwpls** or **ipca** full MSC is performed locally in each interval. When used with **bipls**, **sipls**, and **plsmodel** full MSC is performed on the selected combination of intervals. In a full MSC all variables in the interval(s) are used as the basis.

If you want to test the models on e.g. 1st derivatives of the **X** data simply do the derivation (m-files for this are not included in this toolbox) and use the iToolbox models.

Model structure

The output from iPLS as well as the other methods is a structure array with the following fields:

```
Model =
             type: 'iPLS'
             rawX: [40x926 double]
             rawY: [40x1 double]
         no_of_lv: 10
    prepro_method: 'mean'
      xaxislabels: [1x926 double]
       val_method: 'syst123'
         segments: 5
        intervals: 20
           allint: [21x3 double]
    intervalsequi: 1
         PLSmodel: {1x21 cell}
```

If necessary one can get access to all calculated models, e.g. `Models.PLSmodel{1}` gives the following output on the screen:

```
ans =
    prepro_method: 'mean'
       val_method: 'syst123'
               cv: {[8x1 double] [8x1 double] [8x1 double] [8x1 double]
                                                            [8x1 double]}
           Ypred0: [40x1 double]
            Ypred: [40x1x10 double]
             RMSE: [1x11 double]
             Bias: [1x11 double]
                P: [47x10 double]
                Q: [1 1 1 1 1 1 1 1 1 1]
                W: [47x10 double]
                T: [40x10 double]
                U: [40x10 double]
             bsco: [1x10 double]
           ssqdif: [10x2 double]
```

All models are stored in the same way.

m-files alphabetically

iToolbox directory

bipls

Backward interval PLS: Backwards elimination of non-informative intervals the Leardi way...

biplsdemo

A demo of the biPLS method

biplstable

Presents results from the biPLS method as a table.

contents

The contents file

bipls_dyn

Calculates dynamic biPLS models with a number of intervals defined by the user. The output is used as the input to Leardi's genetic algorithms

intervals

Prints the intervals with variable number and/or wavelength label

ipca

Calculates the interval models based on PCA

ipcademo

A demo of the iPCA method

ipcaloadplot

Makes 2D loading plots of PCa versus PCb for all intervals

ipcascoplot

Makes 2D score plots of PCa versus PCb for all intervals

ipcascoplotall

Makes all 2D score plots up to maxPC for all intervals

ipcavarexp

Makes a bar plot with explained calibration variance for all intervals

ipls

Calculates the interval models based on PLS

iplsdemo

A demo of the iPLS method

iplsplot

Plots results from iPLS analysis in one bar plot

makeClasses

Script file demonstrating how to make classes for coloring in iPCA

makeEntropyIntervals

Calculates indices for entropy intervals based on standard deviations (a la Ralf Torgrip)

makeManualIntervals

Script file demonstrating how to make manual interval splits

makeManualSegments

Script file demonstrating how to make manual segments for cross validation

makeSampleNames

Script file demonstrating how to make samples names for labeling in iPCA

mwpls

Calculates the moving window PLS models

mwplsdemo

A demo of the mwPLS method

mwplsplot

Plots results (RMSECV/RMSEP) from mwPLS analysis

mwplsplot_comp

Plots results (PLSC) from mwPLS analysis

plsmodel

Calculates a PLS model on selected interval(s)

plspredict

Predicts reference values for new X data

plspvsm

Plots predicted versus measured for a combination of one or several intervals

plsrmse

Plots RMSECV/RMSEP as a function of the number of PLS components

sipls

Calculates synergi interval PLS models on combinations of 2, 3, or 4 intervals

siplsdemo

A demo of the siPLS method

siplstable

Lists optimal interval combinations, corresponding RMSECVs/RMSEPs and PLS components

Auxiliary files

The following files are used by the main toolbox files and not further described:

bipls_limit

bipls_vector

bipls_vector_limit

iplsreverse

pca

pls

pls_pre

pls_val
