# Supplementary material for: Dataset of Near-infrared spectroscopy measurement for amylose determination using PLS algorithms
Source: Data Brief. 2017 Oct 6;15:389–96. doi: 10.1016/j.dib.2017.09.077 (PMC5712058; doi:10.1016/j.dib.2017.09.077)
Supplement: Supplementary file 4 — Supplementary material [file mmc4.zip › TOOLBOX/Leardi2004.pdf]

JOURNAL OF CHEMOMETRICS
J. Chemometrics 2004; **18**: 486–497
Published online in Wiley InterScience (www.interscience.wiley.com). DOI: 10.1002/cem.893

Sequential application of backward interval partial least squares and genetic algorithms for the selection of relevant spectral regions

Riccardo Leardi[1]* and Lars Nørgaard[2]
[1]Department of Pharmaceutical and Food Chemistry and Technology, University of Genoa, Genoa, Italy
[2]Chemometrics Group, Department of Food Science, The Royal Veterinary and Agricultural University, Rolighedsvej 30, DK-1958 Frederiksberg, Denmark

Received 13 September 2004; Accepted 27 January 2005

It is nowadays widely accepted that genetic algorithms (GAs) are powerful tools in variable selection and that after suitable modifications they can also be powerful in detecting the most relevant spectral regions for multivariate calibration. One of the main limitations of GAs is related to the fact that when spectral intensities are measured at a very large number of wavelengths the search domain increases correspondingly and therefore the detection of the relevant regions is much more difficult. A modification of interval partial least squares (iPLS), designated backward interval PLS (biPLS), is developed and studied such that it can detect and remove the least relevant regions, thereby reducing the search domain to a size that GAs can handle easily. In this paper the application to two different spectroscopic data sets will be shown: infrared spectroscopic analysis of polymer film additives and determination of the contents of erucic acid and total fatty acids in brassica seeds by near-infrared spectroscopy. The developed method is compared with model performances based on expert selection of variables as well as with results from application of the previously developed GA-PLS method. The sequential application of biPLS and GA-PLS has proven successful, and comparable or better results have been obtained, introducing a more automatic region selection procedure and a substantial decrease in computation time. **Copyright © 2005 John Wiley & Sons, Ltd.**

KEYWORDS: genetic algorithms; backward interval partial least squares; region selection; variable selection; spectroscopy; near-infrared; infrared

1. INTRODUCTION

Good performances of Genetic Algorithms Partial Least Squares (GA-PLS) as a tool for wavelength selection have been reported in previous papers [1–5]. However, study of the behaviour of GA-PLS in such problems has suggested a limitation to no more than 200 variables, since it has been found empirically that a greater number of variables, i.e. a larger search domain, would reduce the capability of obtaining a solution with good predictive ability.

Two different and apparently independent reasons could be at the root of this. The first lies in the fact that the greater the number of variables in the **X** matrix the greater is the probability of finding some chance correlations and therefore of overfitting (this is the reason why a variables/objects ratio ≤ 5 was previously suggested), while the second can be explained by the exponential growth of the search domain (with k variables, $2^k - 1$ combinations are possible).

When dealing with more than 200 wavelengths, the number of variables was previously reduced by applying windows of size n, in such a way that each new variable was the average of the signal intensities at n consecutive wavelengths [1,2]. In the case of spectra with very narrow peaks this approach can be quite dangerous, since some spectral features can be smoothed too much and therefore lose their relevance.

To avoid this problem, an iterative approach was followed in which the least relevant spectral regions (as defined by the frequency of selection by GA-PLS applied to the 'windowed' spectra) were successively removed and therefore the window size could be reduced. This strategy produced good results [3] but required a huge amount of time, since several sets of GA-PLS had to be run (e.g. in the cited paper five GA-PLS calculations were run). Furthermore, at each GA-PLS calculation the decisions about which regions to discard had

*Correspondence to: R. Leardi, Department of Pharmaceutical and Food Chemistry and Technology, University of Genoa, via Brigata Salerno (Ponte), I-16147 Genova, Italy.
E-mail: riclea@dictfa.unige.it
Contract/grant sponsor: Centre for Advanced Food Studies (Major Research Infrastructure).

Copyright © 2005 John Wiley & Sons, Ltd.

to be taken by the user, since it is not possible to fully automate the process.

From the above-cited papers it is observed that the steps preceding the last GA-PLS calculation are used only to remove non-relevant spectral regions rather than as a tool for selecting the relevant wavelengths. It would be an improvement if this relatively easy task could be accomplished by a different method, much faster and in a totally automated way. In this study a new backward interval PLS (biPLS) algorithm is suggested as a fast automatic way to remove non-relevant spectral regions prior to the application of GA-PLS. Two spectroscopic data sets (near-infrared and infrared) are analysed with the suggested combination of methods and compared with model results from expert selection as well as the ordinary GA-PLS approach.

It should be noted that the main purpose of this study is to merge and obtain synergy between two different methods for spectral region selection and that several other methods for region selection have been developed and described in the literature [6–9].

2. THEORY

2.1. Interval partial least squares (iPLS)

Interval PLS (iPLS) [10] calculates local PLS models on equidistant subintervals of the full spectrum region. Its main advantage is to provide an overall graphical illustration of the variation in X relevant to the dependent y-variable. iPLS models are often developed on spectral subintervals of equal width, and the predictive performances of all the local models as well as the global full spectrum model are compared. The model comparison is based on the validation parameter RMSECV/RMSEP (root mean square error of cross-validation/prediction) for a given validation scheme.

Standard iPLS is good in giving a first idea of where the information is, but it is a 'univariate' approach that does not take into account synergism between spectral regions. This was tested in Reference [11], where all combinations of two, three and four intervals were evaluated. This procedure is suboptimal since it is based on investigating a very small part of the very large solution space, and it was clear from the study that more exhaustive search methods such as GAs are more relevant.

iPLS as such, though very fast, is not suited to the goal of removing spectral regions, because it primarily locates the most relevant stand-alone regions, while in the present context there is focus on detecting and removing the least relevant ones.

2.2. Backward interval partial least squares (biPLS)—a new approach

The concept of taking into account intervals, i.e. spectral regions, as blocks of information has been retained and a new algorithm has been developed in which iPLS is applied to the data and then followed by backward elimination, each time eliminating the interval whose removal results in the lowest RMSECV. Note that this is the overall RMSECV computed from the $N-1$ intervals left in the model when iPLS is performed with N intervals (a Venetian blind scheme with five segments was applied). The procedure, designated backward iPLS (biPLS), is either continued until the last interval or can be stopped when the number of retained wavelengths is lower than a predefined threshold, so that a preselected number of variables can be used as an input for GA-PLS.

A major point to be decided in biPLS is the number of intervals. If the number of intervals is too small, then relatively broad spectral areas are analysed each time and therefore the effects of smaller peaks can be lost. On the other hand, if the number of intervals is too high, then the results can be too much on a local scale. Furthermore, a higher number of intervals corresponds to a much longer computation time. As an example, let us suppose we have 1500 variables. If we use 20 intervals, then each interval will be made by 75 variables. This means that, to remove the first interval, 20 PLS models with 1425 variables will be required; the following steps will require 19 models with 1350 variables, 18 models with 1275 variables, and so on. The results of the application of biPLS to several data sets showed that 20 intervals is usually a good value (results not shown), being a reasonable compromise between the two aforementioned effects.

If it has been decided to continue until the last interval, then the evolution of RMSECV as a function of the removed intervals can be followed. This makes it possible to detect the number of intervals leading to the global minimum and therefore the minimum number of wavelengths that have to be retained and passed to GA-PLS.

Two main problems have to be coped with. The first is connected to overfitting. Several times the RMSECV values obtained by the removal of different regions were very similar and therefore the decision about which one has to be eliminated can be different if for example only the compositions of the deletion groups for the cross-validation were different. The solution to this can be found by running biPLS several times, each time with a different composition of the deletion groups.

The second problem depends on the fact that the intervals, by definition, refer to equally spaced spectral regions, without taking into account what is included in each of them. Therefore it can happen that the border between two contiguous intervals falls inside the same spectral feature, e.g. if the main part of a peak is in one interval and its tail in the next one. This would mean that sometimes the spectral regions passed to GA-PLS are not very logical from a spectroscopic point of view. The solution to this problem can be found by running biPLS several times, each time with a different number of intervals, in such a way that the borders between intervals are always different.

A dynamic version of biPLS has therefore been developed. In it the biPLS previously described is run several times, each time with a different composition of the deletion groups (determined by randomizing the order of the samples) and with a different number of intervals (e.g. from 16 to 25). The final output is a plot showing how many times each wavelength has been retained. The wavelengths that have been selected more than a threshold value will be passed to GA-PLS. GA-PLS is then applied on the retained spectral regions with the goal of refining the model.

Copyright © 2005 John Wiley & Sons, Ltd.

2.3. Genetic algorithm (GA)

A GA-PLS specially devoted to wavelength selection has been applied. This algorithm has already been described in References [2,3] and the reader is referred to these papers for more details.

2.4. Software

MATLAB version 6 from MathWorks is used for the calculations, and the iToolbox (including methods for iPLS, biPLS and dynamic biPLS) and GA-PLS Toolbox are both available from http://www.models.kvl.dk.

3. RESULTS AND DISCUSSION

3.1. Polymer films

The combination of dynamic backward iPLS and GA-PLS has been applied to a data set of FT-IR spectra of polymer films in which the amount (ppm) of two additives (B and C) has been measured. The results of the application of iterative GA-PLS on this data set have already been published [3]. The iterative GA-PLS model was at least as good as the model proposed by the spectroscopists in terms of predictive ability, and it was better in terms of parsimony (fewer wavelengths selected) and interpretability (highly significant regions selected). The only drawback of this approach was the long computation time, derived from the fact that five iterations were needed with window sizes of 10, five, four, three and two points respectively.

The spectra have been recorded from 4012 to 401 cm^{-1} (wavelength numbers 1–1873) with a step of 1.93 cm^{-1}, resulting in a total of 1873 wavelengths. The samples were obtained from five production batches, with samples from batches 1–3 being used as calibration set and samples from batches 4 and 5 being used as independent validation set. For additive B there are 42 calibration samples and 28 validation samples, while for additive C there are 109 calibration samples and 65 validation samples. Before the application of GA-PLS a pathlength correction has been applied. Since even under well-controlled situations the film thickness can vary slightly, a correction is made using a polymer peak in the spectrum. The pathlength normalization factor is computed as the average peak height between 2662 and 2644 cm^{-1} (10 data points, wavelength numbers 701–710) minus a baseline value estimated as the average from 2459 to 2442 cm^{-1} (10 data points, wavelength numbers 806–815). The pathlength normalization was computed in this manner because it is known that the peak height of the band at 2662–2644 cm^{-1} (wavelength numbers 701–710) is solely related to the polymer and is thus directly proportional to the film thickness. Figure 1 shows the average spectrum.

Dynamic backward iPLS has been applied with a stop criterion of a maximum of 400 wavelengths. As previously stated, the upper limit for the application of GA-PLS is 200 variables. With less than 400 wavelengths being selected, a very small window of just two consecutive wavelengths will produce less than 200 variables.

3.1.1. *Additive C*

A dynamic biPLS consisting of 30 runs has been performed, with the number of intervals varying from 16 to 25 and with three runs (each with a different composition of the deletion groups) for each number of intervals. The frequency of selections is shown in Figure 2(a).

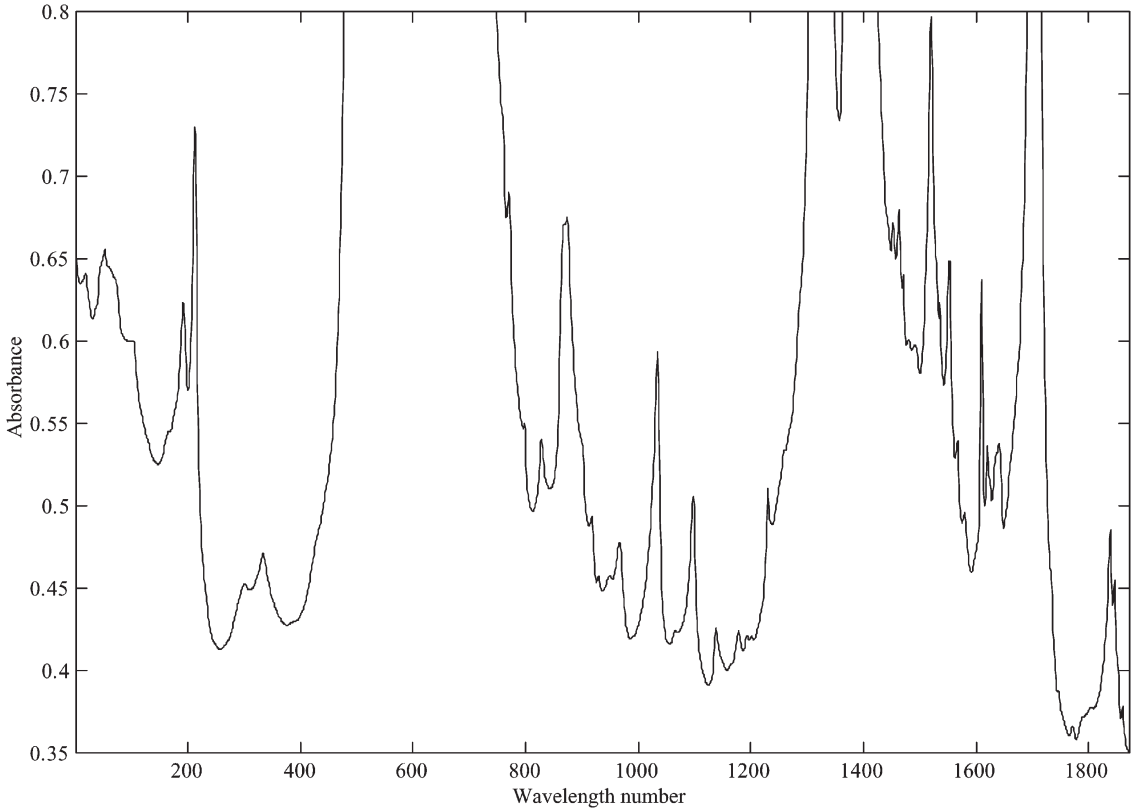

Figure 1. Data set polymer film. Average FT-IR spectrum of 174 samples. Wavelength numbers 1 and 1873 correspond to 4012 and 401 cm^{-1} respectively.

Copyright © 2005 John Wiley & Sons, Ltd.

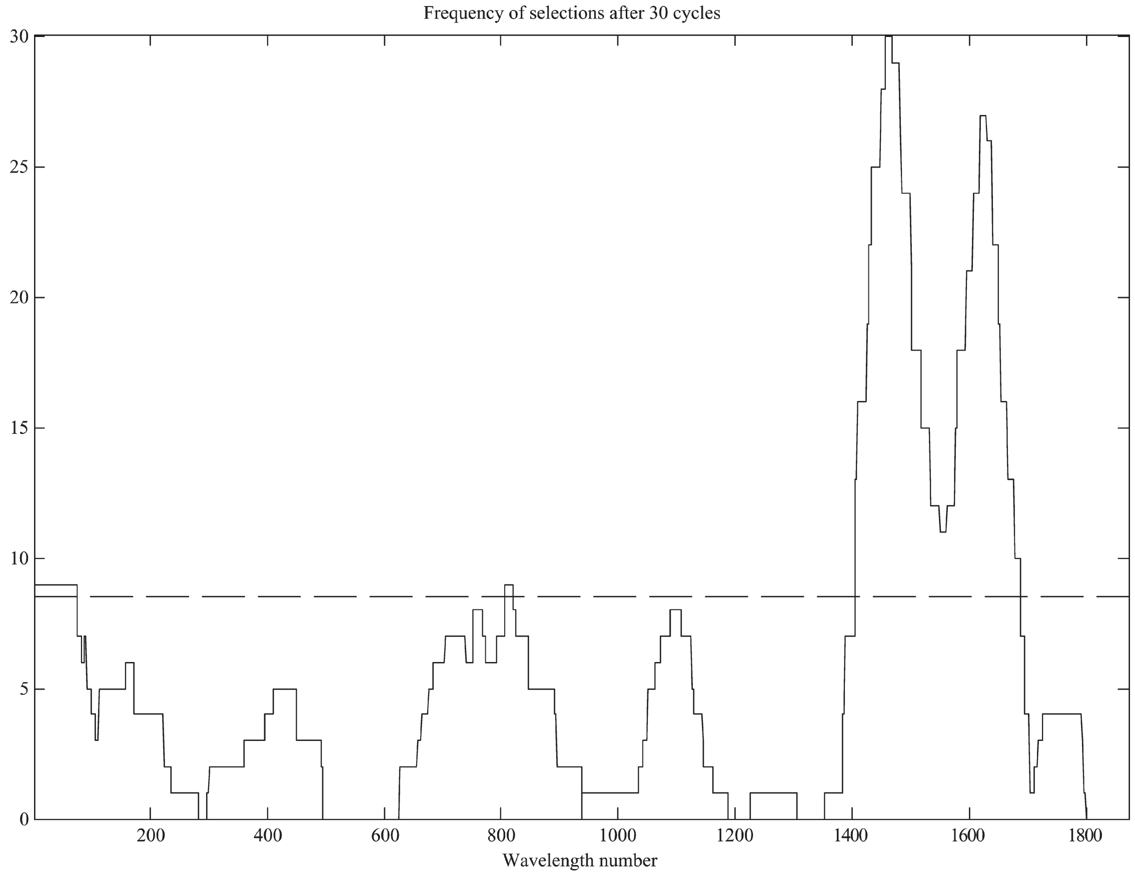

(a)

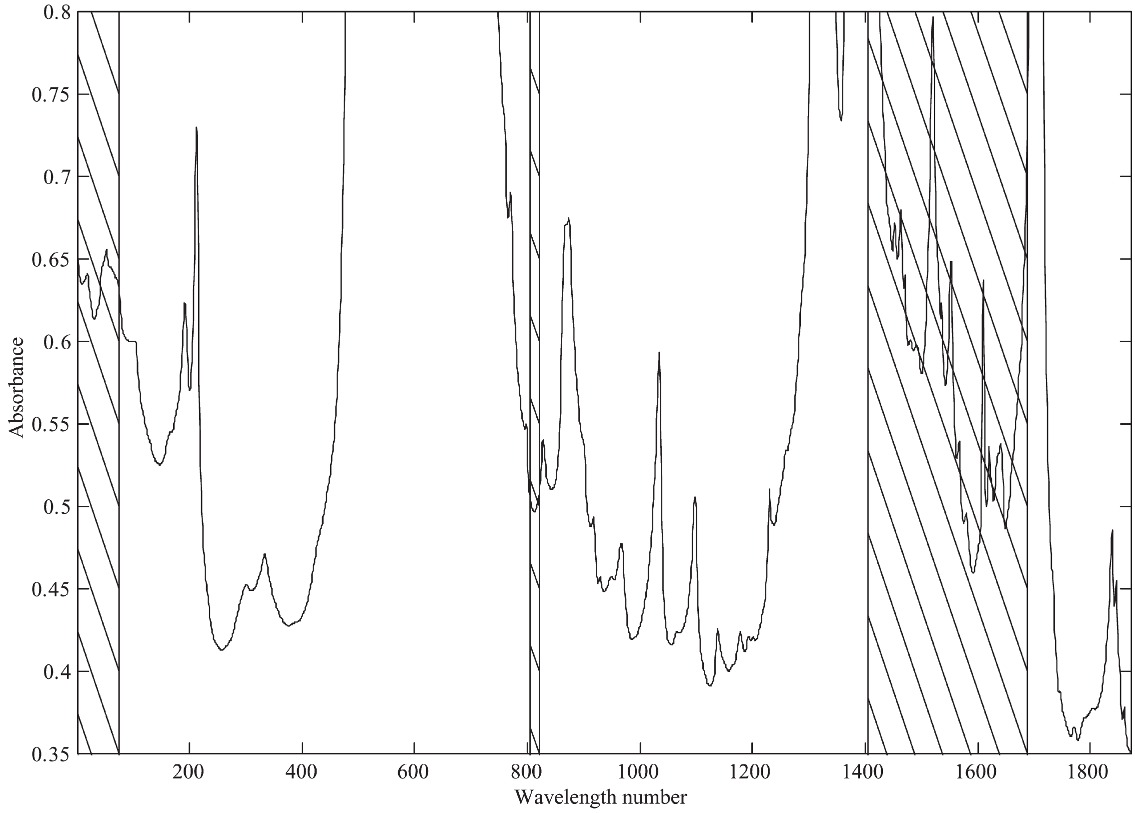

(b)

Figure 2. Continues

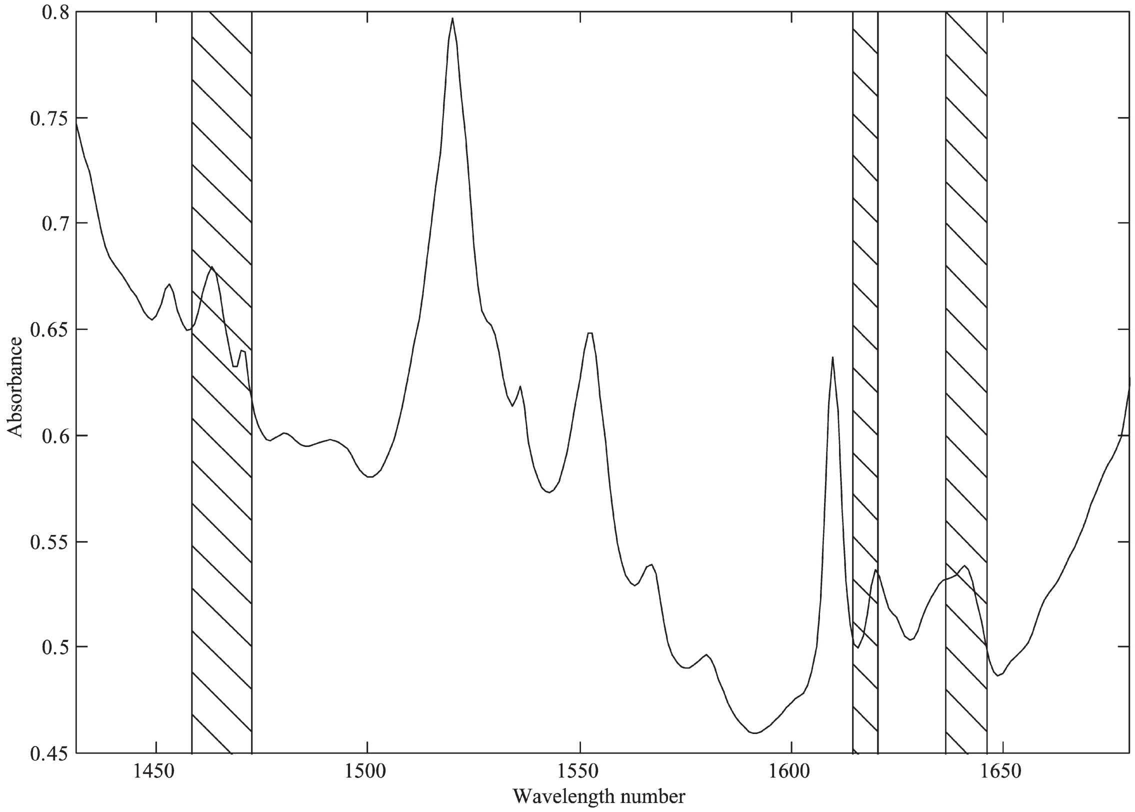

(c)

Figure 2. Data set polymer film, response additive C. (a) Frequency of selections of original wavelengths after dynamic biPLS (30 runs). (b) Location of regions retained by dynamic biPLS. (c) Location of regions selected by GA-PLS applied after biPLS.

Three hundred and seventy-four wavelengths, detecting three regions (wavelength numbers 1–74, 805–820 and 1405–1688), have been retained. The third region contains both the expert-selected region (1615–1651, 37 wavelengths) and the three regions selected by iterative GA-PLS (1459–1472, 1617–1622 and 1633–1646, 34 wavelengths). Figure 2(b) shows the location of the retained regions in the spectrum.

By applying a window size two, the 374 wavelengths have been reduced to 187 variables, on which GA-PLS has been run. The final model selected by GA-PLS was made by 15 variables, corresponding to the following 30 wavelengths, shown in Figure 2(c): 1459–1472, 1615–1620 and 1637–1646. It can be seen that they correspond almost perfectly correspond to the three regions selected by iterative GA-PLS. Of course, also the RMSEP of the independent validation set is very similar (48 vs 47 ppm, with 11 vs 12 components; see Table I). It can be seen that on this response the same results of the cumbersome iterative GA-PLS have been obtained in a much faster and more automated way. The pure computation time for the new algorithm was approximately three times shorter than for iterative GA-PLS. Much more relevant, however, is the time saved by the fact that the new algorithm is almost completely automated, while each iteration of iterative GA-PLS requires subjective judgement by the data analyst.

3.1.2. *Additive B*

A dynamic biPLS with the same structure as for additive C has been performed. The frequency of selections is shown in Figure 3(a). Three hundred and eighty-six wavelengths, detecting four regions (173–314, 1063–1144, 1425–1468 and 1756–1873), have been retained (see Figure 3(b)). Iterative GA-PLS selected the following six regions: 197–206, 263–274, 1093–1104, 1219–1228, 1307–1310 and 1747–1758 (60 variables). It can be seen that the first region selected by biPLS contains the first two regions of iterative GA-PLS and the major part of the region selected by the experts (214–391, 178 variables), the second region of biPLS contains the third region of iterative GA-PLS, and the fourth region of biPLS contains the last wavelengths of the sixth region of iterative GA-PLS. The only discrepancy is between the third region of biPLS and the fourth and fifth regions of iterative GA-PLS.

By applying a window size two, the 386 wavelengths have been reduced to 193 variables, on which GA-PLS has been run. The final model selected by GA-PLS was made by 24 variables, corresponding to the following 48 wavelengths, shown in Figure 3(c): 175–180, 263–268, 1077–1088, 1763–1780 and 1865–1870. The corresponding wavenumbers are given in Table I.

In this case the correspondence with the variables selected by iterative GA-PLS is much lower, since only the second region corresponds to the second region of iterative GA-PLS and falls inside the region selected by the experts. However, the RMSEP of the independent validation set was very similar (49 vs 48 ppm, with 8 vs 6 components). Also on this response the same predictive ability of the cumbersome iterative GA-PLS has been obtained in a much faster and

Copyright © 2005 John Wiley & Sons, Ltd.

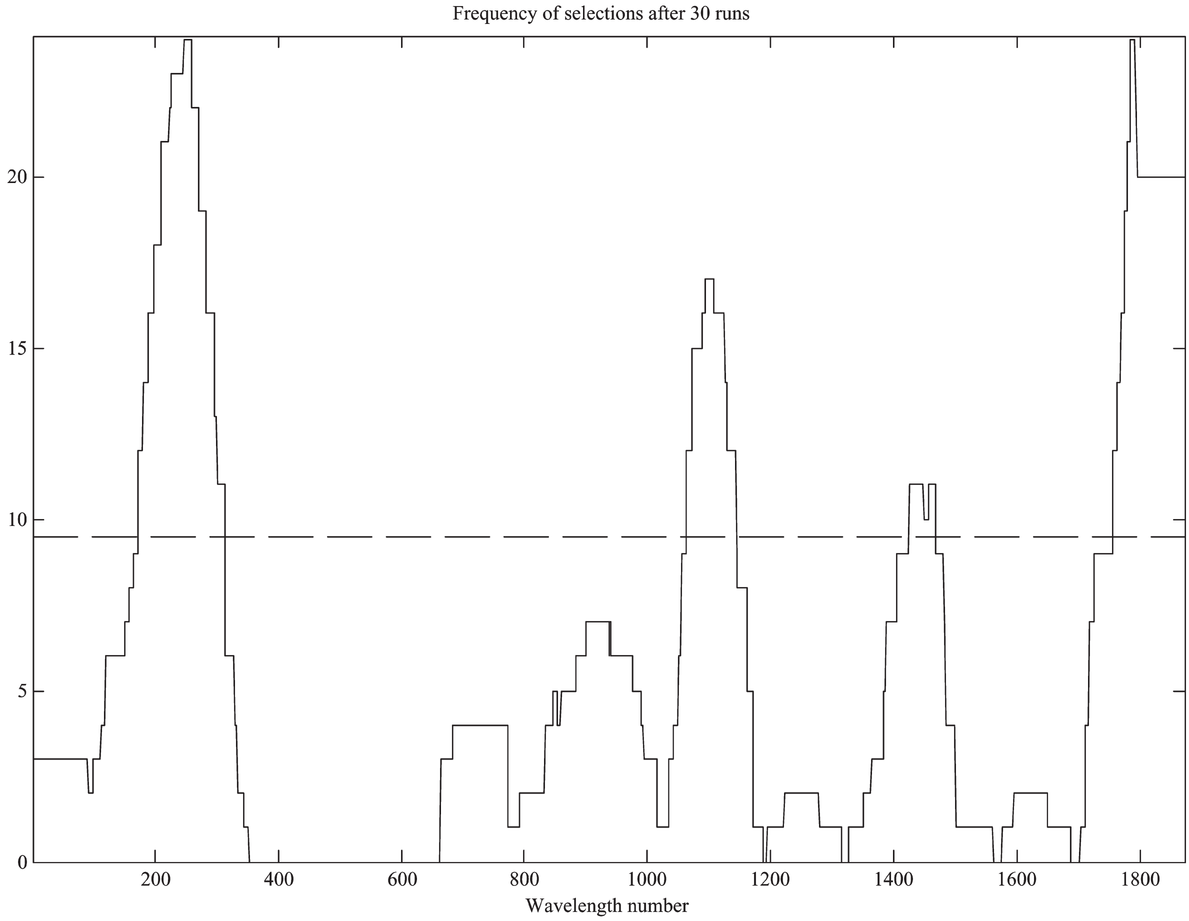

(a)

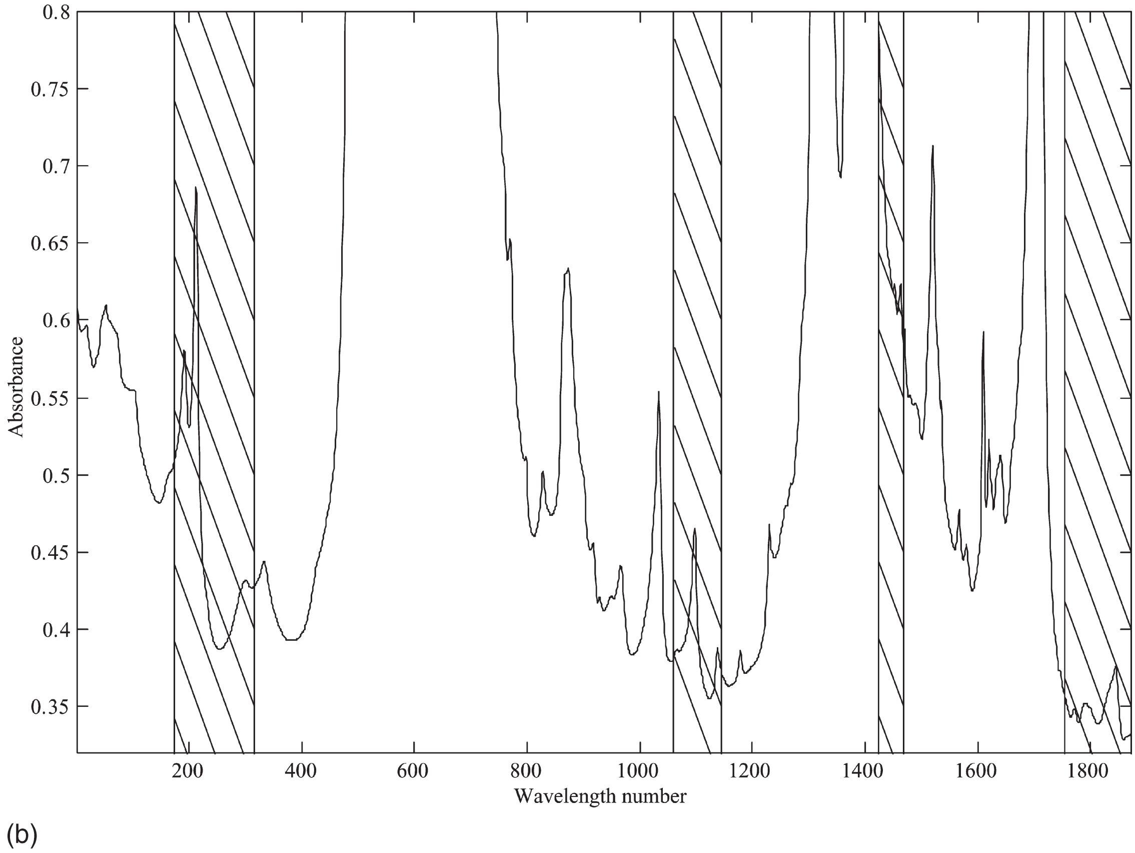

(b)

Figure 3. Continues

Copyright © 2005 John Wiley & Sons, Ltd.

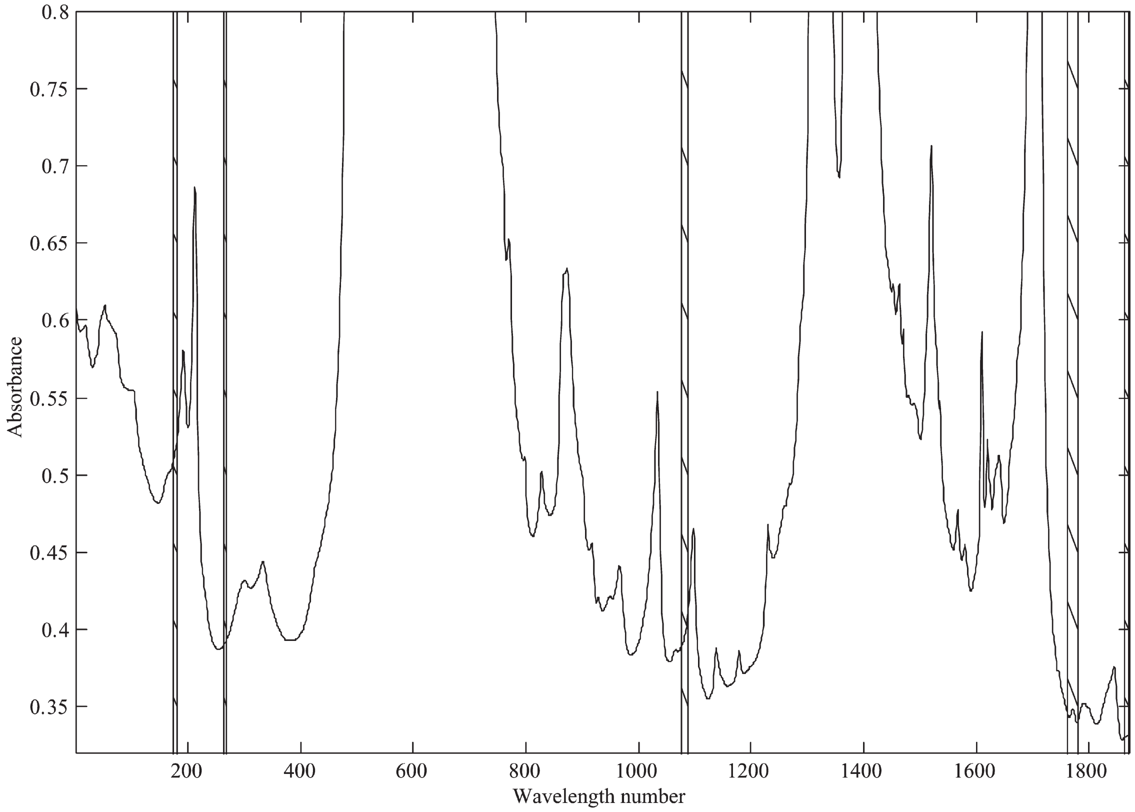

(c)

Figure 3. Data set polymer film, response additive B. (a) Frequency of selections of original wavelengths after dynamic biPLS (30 runs). (b) Location of regions retained by dynamic biPLS. (c) Location of regions selected by GA-PLS applied after biPLS.

more automated way. The time saving for this case is the same as described for additive C.

Table I summarizes the results obtained on the two data sets by the three models: (a) expert, (b) iterative GA-PLS and (c) dynamic biPLS and GA-PLS. It can be seen that the RMSEPs and selected regions obtained with the new method are the same as those obtained by the much more time-consuming iterative GA-PLS; in the case of additive B the predictive ability of both GA-based algorithms is better, though not significantly better, than that of the model suggested by the experts.

Table I. Comparison of selected wavelengths and RMSEP on polymer film data set

	Additive C			Additive B		
Model	Selected wavelengths	Number of wavelengths	RMSEP (ppm) ($n = 65$)	Selected wavelengths	Number of wavelengths	RMSEP (ppm) ($n = 28$)
Expert	1615–1651[a]	37	48	214–391[d]	178	54
Iterative GA-PLS	1459–1472<br>1617–1622<br>1633–1646[b]	34	47	197–206<br>263–274<br>1093–1104<br>1219–1228<br>1307–1310<br>1747–1758[e]	60	48
Dynamic biPLS and GA-PLS	1459–1472<br>1615–1620<br>1637–1646[c]	30	48	175–180<br>263–268<br>1077–1088<br>1763–1780<br>1865–1870[f]	48	49

[a] Corresponds to 899–829 cm^{-1}.
[b] Corresponds to 1200–1175, 895–885 and 864–839 cm^{-1}.
[c] Corresponds to 1200–1175, 899–889 and 856–839 cm^{-1}.
[d] Corresponds to 3601–3259 cm^{-1}.
[e] Corresponds to 3634–3616, 3506–3485, 1906–1884, 1663–1645, 1493–1487 and 644–623 cm^{-1}.
[f] Corresponds to 3676–3666, 3506–3497, 1936–1915, 613–581 and 417–407 cm^{-1}.

Copyright © 2005 John Wiley & Sons, Ltd.

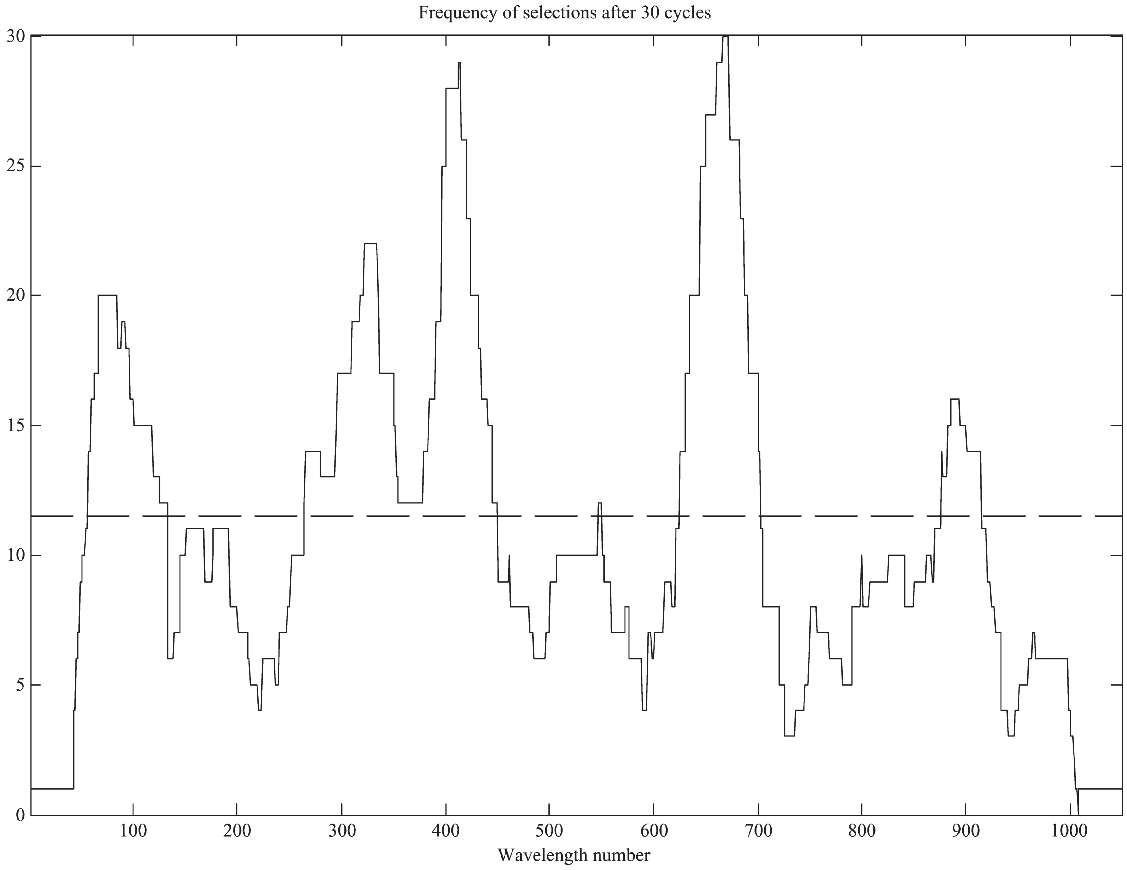

(a)

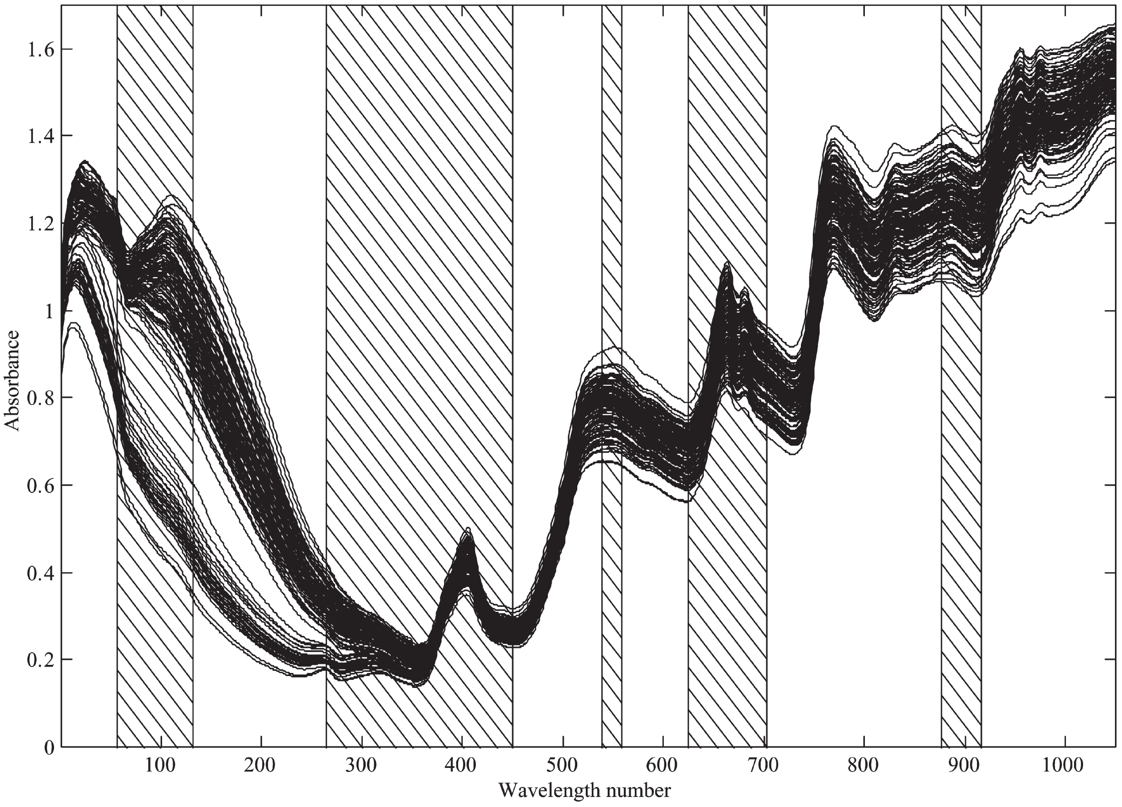

(b)

Figure 4. Continues

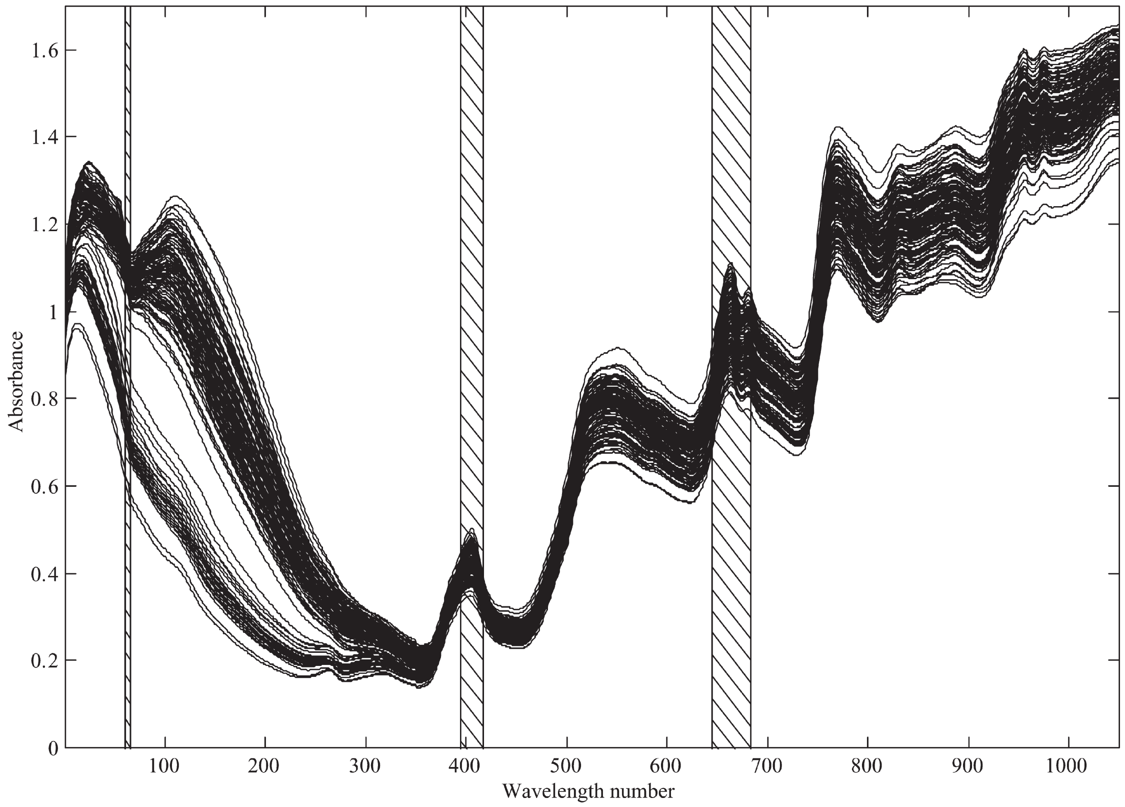

(c)

Figure 4. Data set brassica, response erucic acid. (a) Frequency of selections of original wavelengths after dynamic biPLS (30 runs). (b) Location of regions retained by dynamic biPLS. (c) Location of regions selected by GA-PLS applied after biPLS.

3.2. Brassica seeds

The second data set on which this approach has been applied comes from the Department of Agronomy and Plant Breeding, Institute for Sustainable Agriculture, CSIC, Córdoba, Spain. On 209 samples of Indian mustard (*Brassica juncea* L. Czern. & Coss.), each sample being a seed of different genotype of the plant, 1050 spectral variables were measured with the goal of obtaining a model predictive of the contents of total fatty acids and erucic acid in the seeds [12].

Seed samples were selected to cover the range of chemical variability for total oil and erucic acid contents present in the original collection. Samples were analysed for total oil content (% dry weight) by nuclear magnetic resonance (NMR) in an Oxford 4000 spectrometer (Oxford Instruments, Oxford, UK) validated with the Soxhlet method, and for erucic acid content (% of total fatty acids) by gas–liquid cromatography (GLC) on a Perkin Elmer Autosystem gas–liquid chromatograph equipped with a flame ionization detector (FID). The seeds were scanned in an NIR spectrophotometer (NIRSystems model 6500, Foss-NIRSystems, Inc., Silver Spring, MD, USA) in the reflectance mode, acquiring their spectra at 2 nm intervals over a wavelength range from 400 to 2500 nm (vis + NIR regions).

The samples were divided into a training set (139 samples) and a validation set (70 samples). Splitting was done in such a way that both groups presented similar chemical variability for both chemical components. The RMSEP on the whole spectrum was 4.84% (15 components) for erucic acid and 1.05% (12 components) for total fatty acids.

3.2.1. *Erucic acid*

A dynamic biPLS with the same structure as previously described has been performed. The frequency of selections is shown in Figure 4(a). Four hundred wavelengths, detecting five regions (57–132, 265–450, 539–558, 625–702 and 877–916), have been retained (see Figure 4(b)). From Figure 4(a) it can be seen that the third region was originally very small (547–550); since the original selection was leading to 384 wavelengths, 16 wavelengths have been added to expand this region to stabilize the models.

GA-PLS applied on the whole spectrum, with a window size of six contiguous wavelengths, selected two regions (391–420 and 643–684, see Table II for corresponding wavelengths), for a total of 72 wavelengths, producing an RMSEP of 4.74% (11 components). From Figure 4(b) it can be seen that both these regions are inside the regions selected by dynamic biPLS.

By applying a window size two, the 400 wavelengths from dynamic biPLS have been reduced to 200 variables, on which GA-PLS has been run. GA-PLS selected three regions (61–66, 395–416 and 645–682), for a total of 66 wavelengths (Figure 4(c)), producing an RMSEP of 4.47% (nine components).

Copyright © 2005 John Wiley & Sons, Ltd.

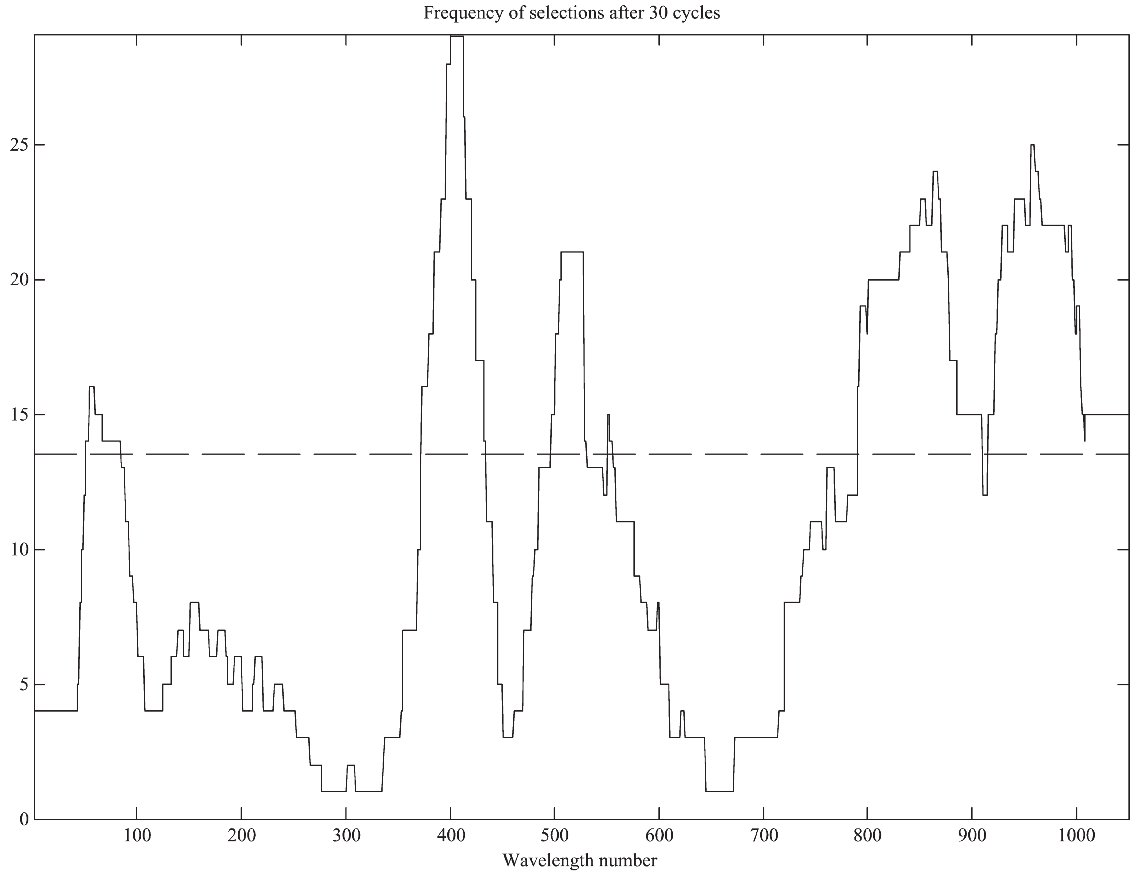

(a)

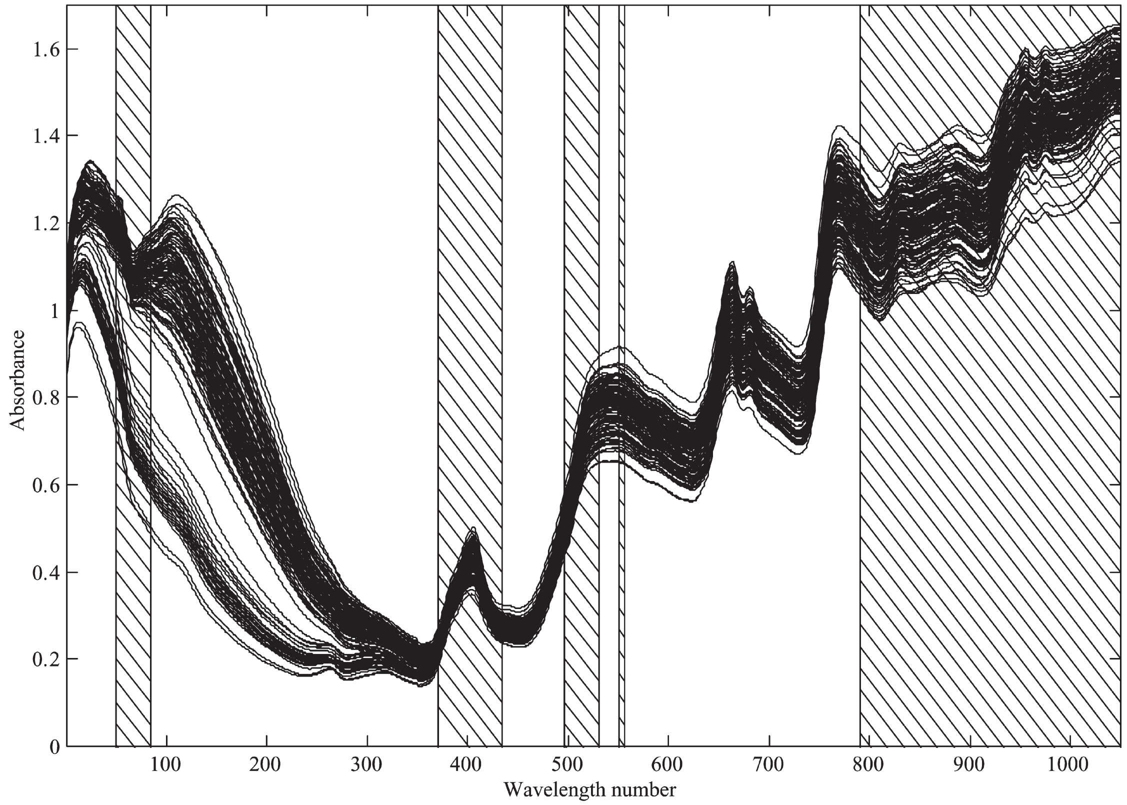

(b)

Figure 5. Continues

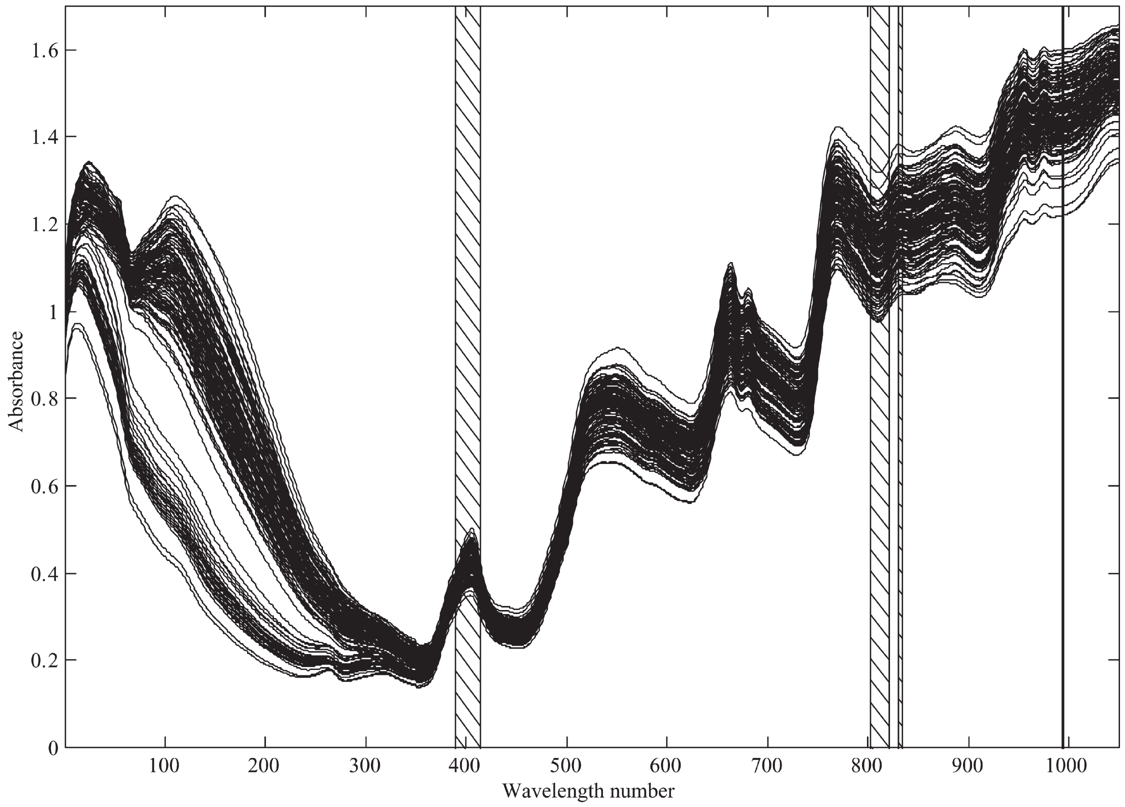

(c)

Figure 5. Data set brassica, response total fatty acids. (a) Frequency of selections of original wavelengths after dynamic biPLS (30 runs). (b) Location of regions retained by dynamic biPLS. (c) Location of regions selected by GA-PLS applied after biPLS.

The comparison of the selected regions shows that the second and the third correspond almost perfectly to the selections made by GA-PLS on the whole spectrum, while GA-PLS on the wavelengths retained by dynamic biPLS detects a third, very tiny region corresponding to a well-defined spectral feature. By adding this third region, a small though not significant improvement in the predictive ability has been obtained for the independent test set.

3.2.2. *Total fatty acids*

A dynamic biPLS with the same structure as previously described has been performed. The frequency of selections is shown in Figure 5(a). Three hundred and ninety-eight wavelengths, detecting five regions (51–84, 371–434, 497–530, 551–556 and 791–1050), have been retained (see Figure 5(a)).

GA-PLS applied on the whole spectrum, with a window size of six contiguous wavelengths, selected three regions (385–420, 583–600 and 991–1002, see Table II for corresponding wavelengths), for a total of 66 wavelengths, producing an RMSEP of 0.96% (six components). From Figure 5(b) it can be seen that, while the first and the third region are inside the regions selected by dynamic biPLS, this is not the case for the second region.

Table II. Comparison of selected wavelengths and RMSEP on brassica data set

	Erucic acid			Total fatty acids		
Model	Selected wavelengths	Number of wavelengths	RMSEP (%) ($n = 70$)	Selected wavelengths	Number of wavelengths	RMSEP (%) ($n = 70$)
GA-PLS	391–420<br>643–684[a]	72	4.74	385–420<br>583–600<br>991–1002[c]	66	0.96
Dynamic biPLS and GA-PLS	61–64<br>395–416<br>645–682[b]	66	4.47	389–414<br>803–820<br>831–834<br>993–994[d]	50	0.99

[a] Corresponds to 1180–1238 nm and 1684–1766 nm.
[b] Corresponds to 520–526, 1188–1230 and 1688–1762 nm.
[c] Corresponds to 1168–1238, 1564–1598 and 2380–2402 nm.
[d] Corresponds to 1176–1226, 2004–2038, 2060–2066 and 2384–2386 nm.

Copyright © 2005 John Wiley & Sons, Ltd.

By applying a window size two, the 398 wavelengths have been reduced to 199 variables, on which GA-PLS has been run. GA-PLS selected four regions (389–414, 803–820, 831–834 and 993–994), for a total of 50 wavelengths (Figure 5(c)), producing an RMSEP of 0.99% (seven components).

It can be seen that the first and the fourth region are subregions of the first and the third region selected by GA-PLS on the whole spectrum, while the second and the third region replace the second region of the previous GA-PLS. The RMSEP is on the same level as the RMSEP obtained by GA-PLS on the whole spectrum and, though one more region has been detected, fewer wavelengths have been selected.

Table II summarizes the results obtained by the two models: (a) GA-PLS and (b) dynamic biPLS and GA-PLS. The sequential application of dynamic biPLS and GA-PLS allowed a greater refinement of the model (windows of two wavelengths compared with the six wavelengths of the original GA-PLS elaboration) with just a marginal increase in the elaboration time.

4. CONCLUSIONS

The sequential application of dynamic backward interval PLS and genetic algorithms proved effective when coping with very complex spectra, i.e. spectra characterized by a high number of variables and/or by narrow peaks. biPLS removes the non-informative regions, thereby significantly reducing the number of variables. The subsequent application of GA-PLS to this reduced domain can lead to very efficient and refined models. This process gives predictive performance results comparable to several, and thereby time consuming, applications of the GA-PLS method as well as to models based on expert region selection. A further advantage is that the proposed method is almost fully automated. A part of future research should focus on comparative studies on the many different ways of performing variable/region selection that are presented in the literature.

Acknowledgements
The authors wish to thank the Centre for Advanced Food Studies (Major Research Infrastructure) for providing funding and access to the chemometric facilities at KVL (Denmark).

REFERENCES

1. Leardi R, Lupiáñez Gonzalez A. Genetic algorithms applied to feature selection in PLS regression: how and when to use them. *Chemometrics Intell. Lab. Syst.* 1998; **41**: 195–207.
2. Leardi R. Application of genetic algorithm-PLS for feature selection in spectral data sets. *J. Chemometrics* 2000; **14**: 643–655.
3. Leardi R, Seasholtz MB, Pell RJ. Variable selection for multivariate calibration using a genetic algorithm: prediction of additive concentrations in polymer films from Fourier transform-infrared spectral data. *Anal. Chim. Acta* 2002; **461**: 189–200.
4. Leardi R. Genetic algorithm-PLS as a tool for wavelength selection in spectral data sets. In *Nature-inspired Methods in Chemometrics: Genetic Algorithms and Artificial Neural Networks*, Leardi R (ed.). Elsevier: Amsterdam, 2003; 169–196.
5. Lestander TA, Leardi R, Geladi P. Selection of near infrared wavelengths using genetic algorithms for the determination of seed moisture content. *J Near Infrared Spectrosc.* 2003; **11**: 433–446.
6. Araujo MCU, Saldanha TCB, Galvao RKH, Yoneyama T, Chame HC, Visani V. The successive projections algorithm for variable selection in spectroscopic multicomponent analysis. *Chemometrics Intell. Lab. Syst.* 2001; **57**: 65–73.
7. Kubinyi H. Evolutionary variable selection in regression and PLS analyses. *J. Chemometrics* 1996; **10**: 119–133.
8. Abrahamsson C, Johansson J, Sparen A, Lindgren F. Comparison of different variable selection methods conducted on NIR transmission measurements on intact tablets. *Chemometrics Intell. Lab. Syst.* 2003; **69**: 3–12.
9. Goicoechea HC, Olivieri AC. A new family of genetic algorithms for wavelength interval selection in multivariate analytical spectroscopy. *J. Chemometrics* 2003; **17**: 338–345.
10. Nørgaard L, Saudland A, Wagner J, Nielsen JP, Munck L, Engelsen SB. Interval partial least-squares regression (iPLS): a comparative chemometric study with an example from near-infrared spectroscopy. *Appl. Spectrosc.* 2000; **54**: 413–419.
11. Munck L, Nielsen JP, Moller B, Jacobsen S, Sondergaard I, Engelsen SB, Norgaard L, Bro R. Exploring the phenotypic expression of a regulatory proteome-altering gene by spectroscopy and chemometrics. *Anal. Chim. Acta* 2001; **446**: 171–186.
12. Leardi R, Armanino C, Font Villa R, de Haro Bailón A. Selection of the NIRS regions predictive of oil and erucic acid contents in samples of *Brassica juncea* by genetic algorithm-PLS. *XVII Congr. Naz. di Chimica Analitica*, 2002; 294.

Copyright © 2005 John Wiley & Sons, Ltd.
