# Supplementary material for: Dataset of Near-infrared spectroscopy measurement for amylose determination using PLS algorithms
Source: Data Brief. 2017 Oct 6;15:389–96. doi: 10.1016/j.dib.2017.09.077 (PMC5712058; doi:10.1016/j.dib.2017.09.077)
Supplement: Supplementary file 4 — Supplementary material [file mmc4.zip › TOOLBOX/Norgaard2000.pdf]

Interval Partial Least-Squares Regression (*i*PLS): A Comparative Chemometric Study with an Example from Near-Infrared Spectroscopy

L. NØRGAARD,* A. SAUDLAND, J. WAGNER, J. P. NIELSEN, L. MUNCK, and S. B. ENGELSEN

The Royal Veterinary and Agricultural University, Food Technology, Chemometrics Group, Department of Dairy and Food Science, Rolighedsvej 30, DK-1958 Frederiksberg C, Denmark

A new graphically oriented local modeling procedure called interval partial least-squares (*i*PLS) is presented for use on spectral data. The *i*PLS method is compared to full-spectrum partial least-squares and the variable selection methods principal variables (PV), forward stepwise selection (FSS), and recursively weighted regression (RWR). The methods are tested on a near-infrared (NIR) spectral data set recorded on 60 beer samples correlated to original extract concentration. The error of the full-spectrum correlation model between NIR and original extract concentration was reduced by a factor of 4 with the use of *i*PLS ($r = 0.998$, and root mean square error of prediction equal to 0.17% plato), and the graphic output contributed to the interpretation of the chemical system under observation. The other methods tested gave a comparable reduction in the prediction error but suffered from the interpretation advantage of the graphic interface. The intervals chosen by *i*PLS cover both the variables found by FSS and all possible combinations as well as the variables found by PV and RWR, and *i*PLS is still able to utilize the first-order advantage.

Index Headings: **Interval PLS; Variable selection; NIR, Principal variables; Forward stepwise selection; Recursively weighted regression; Beer; Extract.**

INTRODUCTION

Full-spectrum regression methods such as partial least-squares regression (PLS) and principal component regression (PCR) have abundantly documented their efficiency within the development of rapid spectral analytical screening methods.[1,2] We have previously applied this approach in exploratory spectral investigations of sugar,[3] pectins,[4] and frying oils[5] employing fluorescence and near-infrared (NIR) as well as Fourier transform infrared and Fourier transform Raman spectroscopy. Chemometricians and data analysts are familiar with the concepts and often favor the use of principal components or latent variables as these aim to represent global orthogonal non-correlated data structures deduced from the highly inter-correlated spectral ensembles. Spectroscopists, on the other hand, usually have a preference for variables or intervals of variables in the original variable space because these represent interpretable chromophores, fluorophores, or vibraphores and because a strict orthogonal decomposition is not realistic. Other important reasons for the development of methods for spectral variable or interval selection are the improvement of models with respect to predictive ability and the possibility for development of very fast instruments including reduction of the production costs for such instruments by employing a few critical regions; e.g., in a filter instrument. A short time of analysis makes the instruments suitable for rapid on-line measurements; e.g., within the area of process monitoring and control. With respect to data reduction, variable selection may also be a realistic method since spectral data contain a high degree of covariance and, as such, large amounts of redundant information. The need for chemometric methods for variable or interval selection where information is optimally preserved is therefore very large.

One of the main advantages in multivariate data analysis and latent variable methods is the possibility of projecting multivariate data into few dimensions in a graphical interface. We propose a new type of graphical output which will enhance the information content for standard multivariate regression methods such as PCR or PLS. The method that we will focus on is a new graphically oriented approach for local regression modeling of spectral data called interval partial least-squares regression (*i*PLS). An NIR spectral data set is investigated, which has proven to give suboptimal solutions in standard full-spectrum PLS applications. The purpose of the interval and variable selection is to optimize the predictive power of PLS regression models and to aid in interpretation. The investigation has the aim of making a comparative study of the prediction performance of selected different methods for selection of manifest variables or intervals of manifest variables compared to the results based on full-spectrum models. In addition to *i*PLS, the principal variables (PV) method as developed by Höskuldsson,[6] forward stepwise selection (FSS) of variables, and a newly presented method called recursively weighted regression (RWR) will be investigated.[7] The results from using these methods will be compared to results from using full-spectrum PLS results. Common to the methods investigated is that they are based on no, or simple, search strategies. Methods based on intensive heuristic search strategies such as genetic algorithms will not be investigated in this paper.[8] An important contribution to the discussion of spectral variable selection was recently given by Spiegelman et al. in their paper on a theoretical justification of wavelength selection in partial least-squares regression.[9] In the literature, the use of principal variables as an alternative to principal components for a single matrix was presented by McCabe,[10] and this topic was also treated by Krzanowski in a study on how to preserve multivariate data structure using principal components analysis

Received 7 June 1999; accepted 25 September 1999.

* Author to whom correspondence should be sent.

0003-7028/00/5403-0413$2.00/0

© 2000 Society for Applied Spectroscopy

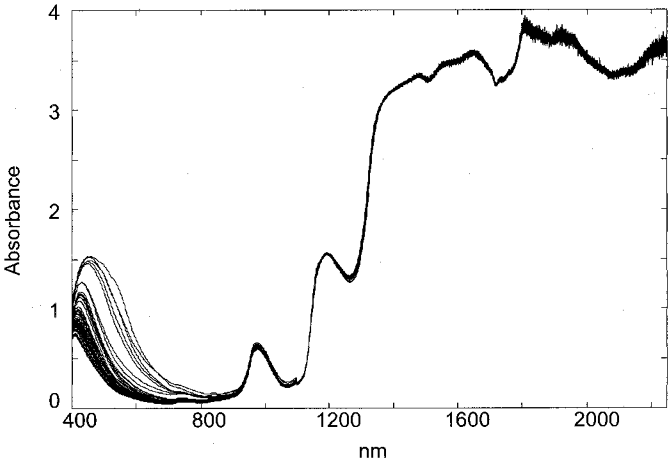

FIG. 1. NIR/visible spectra recorded on 60 beer samples in the wavelength range 400 to 2250 nm; in total 926 variables per sample.

(PCA).[11] Subsequently, Höskuldsson presented a general method capable of dealing with variable selection either in the PCA situation or in the regression situation.[6]

EXPERIMENTAL

Programs. Calculations were performed with Matlab Version 5.2.0 (MathWorks, Inc., Natick, MA) installed with the PLS_Toolbox Ver. 2.0.0b (Wise & Gallagher; Eigenvector Research, Manson, WA) and Unscrambler Version 7.01 (CAMO A/S, Norway). A modified version of a Matlab program made by Agnar Höskuldsson was used for the principal variable selection. The algorithms for *i*PLS, forward stepwise selection, and recursively weighted regression were programmed in the Matlab language by the authors.†

Data Set and Measurement Conditions. We will demonstrate the use and performance of the different variable selection methods by a comparative application to a spectroscopic data set dealing with the determination of the amount of extract from NIR spectra of beers. This data set is an interesting NIR spectral ensemble of 60 beer samples containing a rather large noisy part due to an absorbancy that is too strong, in a region (Fig. 1) dominated by the water component.

Dispersive near-infrared data (including the visual region) at 25 °C were collected with the use of a NIRSystems Inc. (Model 6500) spectrophotometer. The spectrophotometer uses a split detector system with a silicon (Si) detector between 400 and 1100 nm and a lead sulfide (PbS) detector from 1100 to 2500 nm. The NIR/visible transmission spectra were recorded with a 30 mm quartz cell directly on the undiluted degassed beer, and spectral data collected at 2 nm intervals in the range from 400 to 2250 nm were converted to absorbance units.

Original extract concentration is an important quality parameter in the brewing industry, indicating the substrate potential for the yeast to ferment alcohol and serving as a taxation parameter. Original extract concentration was determined by Carlsberg A/S in the range of 4.23–18.76% plato. The data were sorted by extract value, and an independent test set was constructed by selecting every third sample of this full set. There are thus two data sets: one for calibration (40 samples) and one for estimation of prediction error (20 samples). It is assumed that overfitting will be revealed by the independent test set.

† The *i*PLS algorithm including the optimization module and the NIR data set studied in this work is available from our Web site: http://www.mli.kvl.dk/foodtech/special/specials.htm.

CHEMOMETRIC THEORY

Partial Least-Squares Regression. Partial least-squares regression is a predictive two-block regression method based on estimated latent variables and is applied to the simultaneous analysis of two data sets (e.g., spectra and physical/chemical tests) on the same objects[12] (e.g., beer samples). The purpose of the PLS regression is to build a linear model enabling prediction of a desired characteristic ($\mathbf{y}$) from a measured spectrum ($\mathbf{x}$). In matrix notation we have the linear model $\mathbf{y} = \mathbf{Xb}$ where $\mathbf{b}$ contains the regression coefficients that are determined during the calibration step, and $\mathbf{X}$ is the matrix of collected spectra. PLS was first applied to evaluate NIR spectra by Martens and Jensen in 1983,[1] and is now used routinely in academia and industry to correlate (rapid) spectroscopic measurements with related chemical/physical data.

Interval PLS. Interval PLS is an in-house developed interactive extension to PLS, which develops local PLS models on equidistant subintervals of the full-spectrum region. Its main force is to provide an overall picture of the relevant information in different spectral subdivisions, thereby focusing on important spectral regions and removing interferences from other regions. The sensitivity of the PLS algorithm to noisy variables is highlighted by the informative *i*PLS plots.

Interval PLS models are developed on spectral subintervals of equal width, and the prediction performance of these local models and the global (full-spectrum) model is compared. The comparison is mainly based on the validation parameter RMSECV (root mean squared error of cross-validation), but other parameters such as r^2 (squared correlation coefficient), slope, and offset are also evaluated to ensure a comprehensive model overview. Sample and/or measurement abnormalities (outliers) as detected by PLS inner relation plots should generally be removed prior to the application of *i*PLS.

Models based upon the various intervals ($\mathbf{X}_{\text{interval}}$) usually need a different number of PLS components than do full-spectrum models to catch the relevant variation in $\mathbf{y}$. This condition is caused by the varying amount of y-correlated information carried by the interval variables (the larger the spectral interval, the greater the number of substances that are likely to absorb/interfere) and is also related to the noise/interference carried by the variables. However, the selected model dimension has to be common to all the local models in order to make a comparison possible. In order to favor the "best" spectral region, it is natural to let the simplest interval model (i.e., the one with the smallest number of PLS components) guide the selection of the model dimension. A fair comparison of the global and local models requires that the global and local model dimensions be selected separately.

Simple Optimization of the Best Interval from Equidistant iPLS. There is a minimal probability for hitting the

optimal interval with the equidistant subdivisions. A more optimal interval might be found by carrying out small adjustments in the interval limits. The optimization performed consists of the following steps: (1) interval shift; (2) changes in interval width: two-sided (symmetrical), one-sided (asymmetrical, left), or one-sided (asymmetrical, right). Each step is initiated with the optimal interval limits from the previous step. The interval limits are changed one variable at a time and evaluated by the RMSECV provided by application of PLS regression to the interval; this approach works in practice but could be done more elegantly.

Principal Variables. Principal variables is a method for selection of a limited number of original variables (e.g., wavelengths) that describe, as much as possible, the variance in the data matrix (spectra) or, alternatively, covariance in the matrix with a vector with a desired characteristic (chemical/physical measurement).[6] The PV method is initiated by finding the variable (wavelength) that co-varies most with the **y** vector (physical/chemical measurement). This variable is the first principal variable. The original spectral data matrix is then reduced (orthogonalized) with respect to the first principal variable. Then the next covariant variable in the reduced data matrix is selected, and this procedure is followed until the wanted number of principal variables has been calculated. The result of the PV selection is a limited number of the original variables (e.g., wavelengths), while PLS selects latent factors based on information from all original variables. The PV method also works on a single data matrix, in which case the method will search for columns that describe the largest variation; i.e., the method is a general tool for variable selection.

Forward Stepwise Selection of Variables. Forward stepwise selection is a most simple and pragmatic search method in which subsequent variables are selected stepwise by their capability to improve a multiple linear regression (MLR) model. First, all spectral variables are tested individually in univariate linear regression models with extract concentration as the dependent variable. All these models are test set validated, and the variable with the lowest RMSEP (dependent test set) is chosen. Next, all two-variable MLR models are investigated on the basis of the chosen variable in combination with all the remaining variables (one by one). All these models are also test set validated, and the variable that (in combination with the first chosen variable) gives the lowest RMSEP (dependent test set) is chosen. This procedure is continued until the RMSEP (dependent test set) increases by the introduction of a new variable. In the FSS case, a dependent test set is chosen to evaluate the selection of new variables, since an evaluation procedure based on cross-validation leads to severe overfitting.[12,13]

Recursively Weighted Regression. This method is based on an recursive re-weighting of the independent variable block (**X**) by the regression vector **b** calculated from a PLS regression model between **X** and **y**: $\mathbf{x}^i_{n+1} = \mathbf{x}^i_n * b^i_n$, $i = 1$ to number of variables, where b^i_n is the ith element in the PLS regression coefficient vector ($\mathbf{b}_n$) of step number n, and $\mathbf{x}^i_n$ is the ith column of $\mathbf{X}_n$.[7] The algorithm is started with a standard PLS model between $\mathbf{X}_1$ (equal to **X**) and **y**, giving $\mathbf{b}_1$. The re-weighting is repeated 50 times ($n = 1$ to 50) in the calculations presented in this paper in order to ensure that a final solution has been reached. The result is a regression vector $\mathbf{b}_{50}$ that contains only ones and zeros (this binary result is a direct output from the RWR algorithm; i.e., no rescaling of the final regression vector is performed). In the simple case, the number of variables selected (i.e., variables with a corresponding regression coefficient of one) corresponds to the number of latent factors chosen in the original PLS model. This is not the case in more complicated situations. This simple method, which combines multivariate regression and variable selection, has not yet been thoroughly investigated but certainly deserves more attention.

Error Measures. The root mean square error in combination with the correlation coefficient (r) is used as a measure of how a given model performs. RMSE is defined as follows:

$$\mathrm{RMSE} = \sqrt{\frac{\sum (y_{\mathrm{pred}} - y_{\mathrm{ref}})^2}{N}}$$

where y_{pred} is the predicted value, y_{ref} is the laboratory-measured value, and N is the number of samples.

RMSEC is RMSE calculated from the calibration samples, i.e., a measure of fit. RMSECV is calculated from the cross-validated samples, and RMSEP is calculated from the independent test (or prediction) set.[12,13] Correspondingly, r_{cal}, r_{cv}, and r_{pred} are the correlation coefficients for these three situations.

RESULTS AND DISCUSSION

All models are developed on the basis of NIR/visible spectra (**X**) and the response variable extract concentration (**y**). The spectra are shown in Fig. 1. Both mean-centered and autoscaled X data[12] are tested, and all the models developed are validated by segmented cross-validation.[12,13] Five segments are used and they are selected systematically among the 40 calibration samples; i.e., in CV segment number one, the samples 1, 6, 11, 16, 21, 26, 31, and 36 are represented. RMSECV is the parameter governing the variable selection for all tested methods; i.e., the set of variables chosen for a given method is the set that gives the lowest RMSECV among the combinations tested with that method. RMSEP is an estimate of the prediction error based on 20 samples, and its value also reveals whether there are problems with overfitting for some of the methods. In Table I all results on NIR spectral ensemble are compiled.

PLS Full-Spectrum Results. Mean-centered and autoscaled full-spectrum PLS results indicate unstable models when the three RMSE values in Table 1 are compared. Both models are suboptimal, and for the mean-centered model two local minima are seen before the global one at nine PLS components. The improvement in RMSECV when going from five PLS components to nine PLS components is negligible for the model based on autoscaled data (not shown).

***i*PLS Results.** In this section focus is on the situation where the data are autoscaled to provide uniform variance over the entire spectral range (according to the comments made above) and divided into 20 subintervals to show how *i*PLS works. Figure 2 shows y-residual variance characteristics: one for the full-spectrum model and one

TABLE I. **Results for NIR on beer samples when using different chemometric methods for variable selection.**

Method	Preprocessing	# PCs	# Variables	Interval (nm)	RMSEC	r_{cal}	RMSECV	r_{cv}	RMSEP	r_{pred}
PLS	Auto	9	926	400–2250	0.001	1.000	0.80	0.948	0.40	0.993
	Mean	9	926	400–2250	0.005	1.000	1.31	0.849	0.73	0.961
*i*PLS	Auto	4	46	1228–1318	0.10	0.999	0.15	0.998	0.20	0.997
20 intervals	Mean	4	46	1228–1318	0.12	0.999	0.16	0.998	0.21	0.997
*i*PLS	Auto	3	15	1270–1298	0.24	0.995	0.30	0.992	0.21	0.997
60 intervals	Mean	3	15	1270–1298	0.24	0.995	0.31	0.992	0.22	0.997
*i*PLS	Auto	4	48	1228–1322	0.10	0.999	0.13	0.999	0.18	0.998
optimized[a]	Auto	2	49	1202–1298	0.11	0.999	0.15	0.998	0.17	0.998
PV	Auto	MLR	2	1326, 1184[b]	0.21	0.996	0.24	0.995	0.14	0.999
	Mean	MLR	3	440, 536, 1322[b]	0.41	0.986	0.52	0.977	0.34	0.991
RWR	Auto	2[c]/MLR	2	1184, 1326	0.21	0.996	0.24	0.995	0.14	0.999
	Auto	3[c]/MLR	3	1184, 1320, 1950	0.18	0.997	0.21	0.996	0.15	0.999
	Mean	3[c]/MLR	3	1326, 2234, 2246	0.38	0.988	0.44	0.983	0.37	0.991
FSS	None	MLR	2	1326, 1134[b]	0.16[d]	0.998[d]	0.18[d]	0.997[d]	0.17	0.998
All comb.[e]	None	MLR	2	1128, 1314	0.15	0.998	0.16	0.998	0.20	0.997

[a] Results after optimization based on a 20 interval subdivision.
[b] The variables are found in the written order.
[c] Number of PLS components used in the recursive latent models.
[d] Test set validated with a calibration set of 20 samples and a dependent test set of 20 samples. The RMSECV error corresponds to a dependent test set error, while the RMSEP error is the independent error.
[e] All possible two-variable combinations are tested; in total 428 275 models.

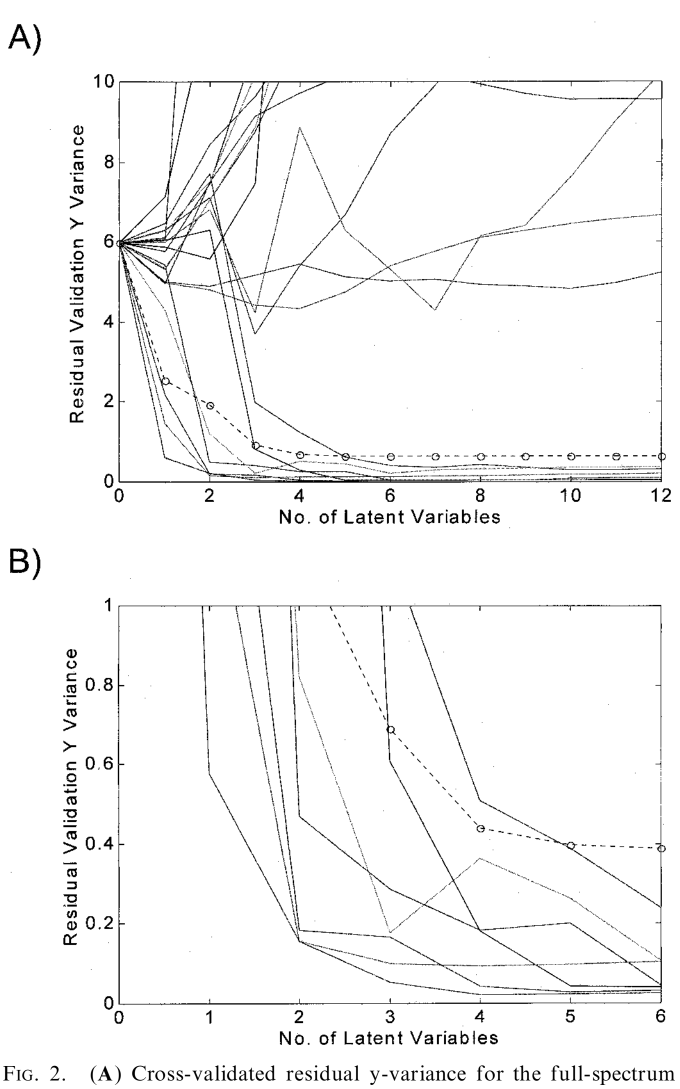

FIG. 2. (**A**) Cross-validated residual y-variance for the full-spectrum model (-o–o) and 20 local models as a function of number of PLS components. (**B**) Enlargement of **A** to show the first minimum at four PLS components for interval 10.

for each of the 20 spectral subdivisions. From Fig. 2 both the local and the global model dimensions are selected. Variance characteristics approaching the abscissa represent promising local models describing most of the systematic variance in the spectral data. In this case there are seven such models, and three of these differ from the rest in having a significant y-residual variance reduction for the very first latent variable. Four PLS components are appropriate for the local models, and in contrast nine PLS components are optimal for the full-spectrum PLS model based on autoscaled data. Figure 3, which demonstrates the central *i*PLS plots, shows expected prediction error (RMSECV) for 20 interval models (bars) and for the full-spectrum model (line) plotted together with a normalized mean spectrum. In Fig. 3A, one PLS component is used in the interval models and for Figs. 3B, 3C, and 3D the number is two, three, and four, respectively. The full-spectrum model (line) is based on nine PLS components in all four plots. It appears from Fig. 3A that only one interval model (number 10) with one latent variable can compete with the full-spectrum model using nine PLS components. However, when two to four (optimal) PLS components are used, several interval models surpass the full-spectrum model.

Interval number 10 (46 variables) was chosen for further optimization: (1) an interval shift of 30 variables to each side was performed, followed by (2) changes in interval width from a chosen minimum of 30 to a chosen maximum of 110 variables [first two-sided symmetrical optimization, then one-sided asymmetrical (left) and one-sided asymmetrical (right) optimization]. The optimization results in an interval in the range 1228–1322 nm (see Table I) with the use of four PLS components. A thorough optimization procedure might include different numbers of PLS components, since a smaller interval might be modeled by a lower number of PLS components. This approach is illustrated by the results from a two-PLS-components solution given in Table I.

Furthermore, the effect of the number of start intervals can be optimized to see how this number influences the

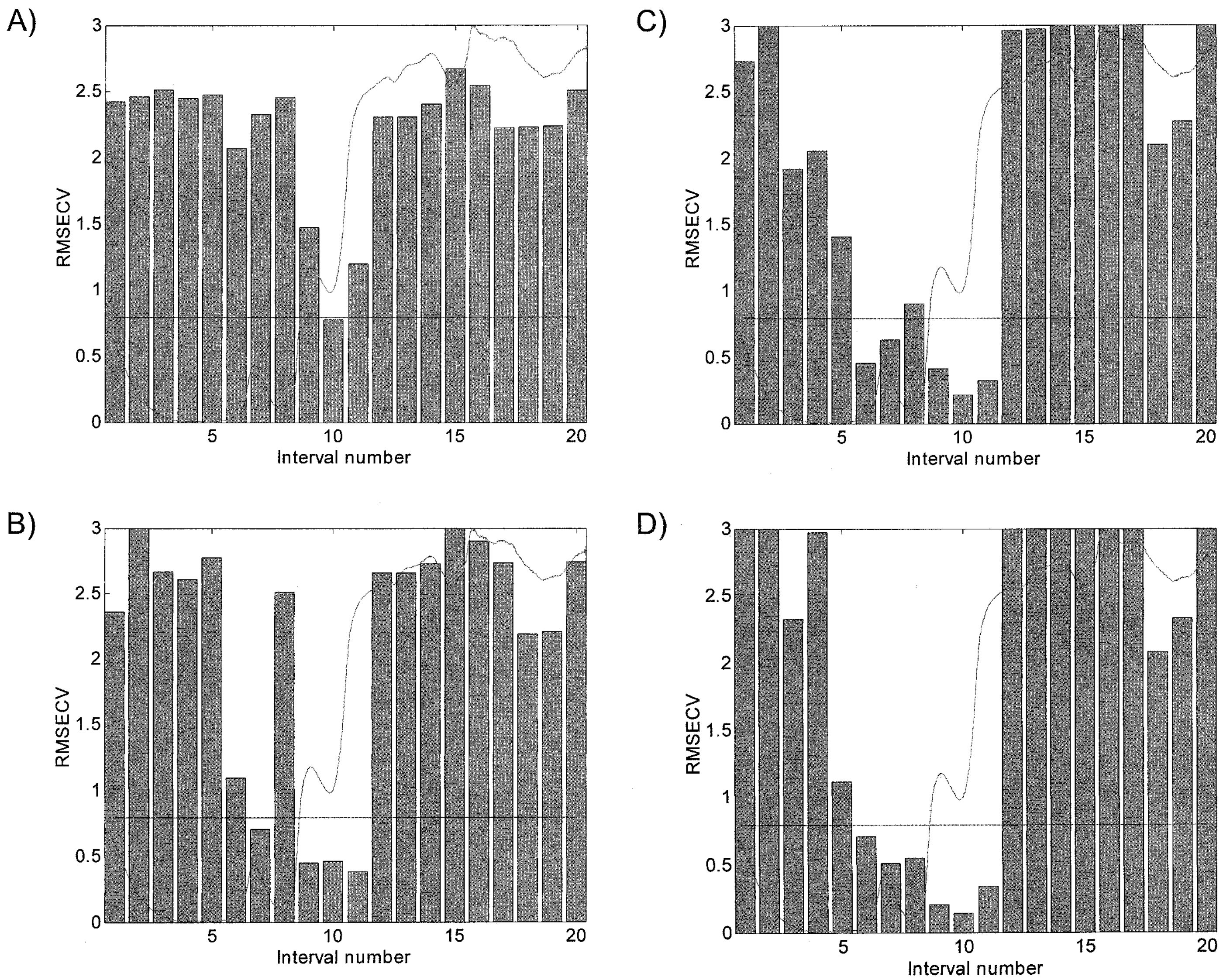

FIG. 3. Cross-validated prediction performance (RMSECV) for 20 interval models (bars) and for full-spectrum model (line) plotted together with the normalized mean spectrum. The interval models used one (**A**), two (**B**), three (**C**), and four (**D**) PLS components, respectively, in the four plots, while the full-spectrum model was kept at nine PLS components (autoscaled data).

results. In Table I results from using both 20 and 60 intervals are shown. The results from using 20 intervals (46 variables in each model) seem more robust than those from the use of 60 intervals (15 variables in each model). Different numbers of intervals can be tested in new applications to see how the information changes with respect to the variables included in the modeling.

Results from Other Methods (PV, FSS, RWR, and All Possible Combinations). Results from principal variables, forward stepwise selection, and recursively weighted regression selection of variables are shown in Table I. Also the optimal result from all possible two-variable combinations (=428 275) are shown. All selected variables are based on the lowest value of RMSECV.

Discussion. Comparing Figs. 1 and 3D, we see that both the noisy region from 1400 to 2250 nm and the systematic visual region from 400 to 800 nm are found to be of no relevance when building correlation models to the original extract. In this way *i*PLS gives an overview of the spectral data and reveals the interesting parts of the spectrum, helping in chemical interpretation. In this case the transparent spectral NIR region between the visual region (400–800 nm) and the NIR region where the strongly absorbing O–H vibrations of the water begin to appear (from 1400 nm and up) holds the predictive performance with respect to the original extract measurement. Except for the second overtone of the O–H stretch at ~ 970 nm, this NIR region is dominated by C–H and N–H stretching overtones. It is seen that this data set without *a priori* knowledge may cause severe troubles for the PLS algorithm. The experienced spectroscopist would remove the noisy spectral region prior to PLS calibration, but this investigation aims at illustrating the usefulness of variable or interval selection when (PLS) calibration is performed on new data to which no prior knowledge is available, or when PLS is applied to data sets which are too large and/or inhomogeneous for standard exploratory PLS investigation.

From Table I we see that none of the full-spectrum models perform well compared to the selection methods.

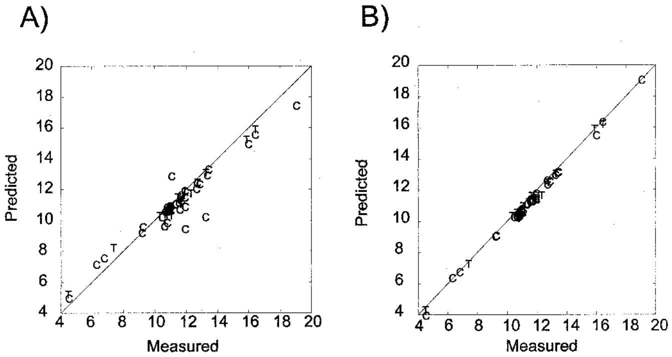

FIG. 4. (**A**) Predicted vs. measured plot for the full-spectrum PLS model with nine PLS components on autoscaled data. (**B**) Corresponding plot for the best interval (1228–1318 nm) from an *i*PLS model with 20 subdivisions without further optimization. In both plots, C denotes cross-validated predictions and T denotes the independent test set predictions.

There is no systematic trend between the RMSEC, RMSECV, and RMSEP values, which is to be expected for a robust model. This observation is due to the rather noisy region in the range 1400–2250 nm, which spoils the full-spectrum PLS model. Also mean-centered PV and RWR fail to find a suitable combination of variables. Compared with all possible two-variable combinations, the methods of *i*PLS, PV, and RWR work well on autoscaled data; *i*PLS also works well on mean-centered data. FSS is scaling independent, and it is the only variable selection method that finds a pair of variables that compares very well to the result found by all possible combinations of two variables. Both the RWR and PV methods select a different combination of variables that is almost alike for the two methods. The intervals chosen by *i*PLS cover both the variables found by FSS and all possible combinations as well as the variables found by PV and RWR. The RWR method is robust with respect to the number of PLS components chosen as a starting point (two or three) for the PLS models when using autoscaling. This observation is reflected in the regression coefficients of the RWR model based on three PLS components resulting in a three-variable multiple linear regression model with the following regression coefficients: $(b_0, b_1, b_2, b_3) = (83.94, 84.06, -88.94, -2.87)$. The regression coefficient b_3 of the noisy variable at 1950 nm has a low numerical value compared to b_0, b_1, and b_2 when considering the absorbance values at the three variables, indicating that the first two variables (1184 and 1320 nm) are sufficient for building a regression model; b_0 is the offset in the regression model.

The optimized PLS model does not give a decrease in RMSECV, reflecting that it is difficult to optimize the actual model further. The decrease from 0.15 to 0.13 is not significant when we take into account the uncertainty of the original extract measurement (estimated to be ~0.02–0.04% plato). This observation is supported by the fact that the optimized interval (1228–1322 nm) is almost exactly the same as the first chosen interval (1228–1318 nm), reflecting a chance improvement. Furthermore, by using the interval 1202–1298 it is possible to obtain comparable RMSECV results with only two PLS components.

In Fig. 4 the predicted vs. measured plots for a full-spectrum model and the best interval model (without optimization) are given to show the significant decrease in RMSECV and RMSEP. In this case the interval model is superior to the full-spectrum model. It should be stressed that knowledge of the reproducibility of the spectral measurements (not available here) might be used in preprocessing of the spectral data so that the noisy part of the spectrum is down-weighted in a PLS analysis.

Furthermore, it should be emphasized that especially minimalistic variable selection reduces the power of multivariate outlier control and increases the influence of spectral noise. The trade-off between the measurement of few variables and a reduced quality of outlier detection must be evaluated for each application, and the optimal choice might be different depending on the actual spectroscopic technique.

Finally, it should be mentioned that the *i*PLS method was preliminarily tested on an NIR data set of pectins with different degrees of esterification. With 50 *i*PLS intervals, one interval with two PLS components improved the performance of a four-PLS-component full-spectrum model by a factor of 2 with respect to RMSECV.[14] All other intervals gave higher RMSECV values compared to the RMSECV of the full-spectrum model.

CONCLUSION

It might be very useful to select variables or intervals of variables from spectroscopic data ensembles. In this paper a new graphically oriented local modeling approach (*i*PLS) is described and compared to three different variable selection methods by evaluation on a near-infrared spectroscopic data set. For these data it has been shown that *i*PLS is an attractive method in providing an overview of interesting spectral areas which could be selected. The results from using *i*PLS are comparable to the other effective methods tested, but the main contribution from using *i*PLS is the graphic output giving an overview of the spectral data. For specific selection of variables, forward stepwise selection proved to be a good alternative, while methods such as recursively weighted regression and principal variables work well in some cases, depending on the preprocessing of the data. Basically, *i*PLS has proven to represent a sound compromise between data reduction and spectral localization and yet being able to utilize the first-order advantage.

Further research on *i*PLS might include an investigation of all possible combinations of the selected intervals in order to investigate the synergy between different spectral regions. If the number of intervals chosen is less than approximately 20–30, it is possible to evaluate all possible interval combinations depending on how much computer time one can spend. Work is also currently underway with respect to improving and generalizing the interval optimization.[15]

ACKNOWLEDGMENTS

Agnar Höskuldsson, Rasmus Bro, and Carsten Ridder are acknowledged for inspiring discussions on the topic treated in this paper. The investigation is sponsored by Centre for Advanced Food Studies (LMC) with funds for Søren Balling Engelsen and by funds to Professor Lars Munck from the Danish Research Councils 13-4804-1 (agriculture) and 16-5180-1 (technology) and Centre for Predictive Multivariate Process

Analysis. Carlsberg are thanked for making available the analyzed beers including determination of extract concentration.

1. H. Martens and S. A. Jensen, "Partial Least Squares Regression: A New Two Stage NIR Calibration Method", in *Progress in Cereal Chemistry and Technology,* J. Holas and J. Kratochvil, Eds. (Elsevier, Amsterdam, 1983), pp. 607–647.
2. K. H. Norris, *Near Infrared Technology in Agricultural and Food Industries* (American Association of Cereal Chemists, St. Paul, Minnesota, 1987).
3. L. Munck, L. Nørgaard, S. B. Engelsen, R. Bro, and C. A. Andersson, Chemom. Intell. Lab. Syst. **44,** 31 (1998).
4. S. B. Engelsen and L. Nørgaard, Carbohydrate Polym. **30,** 9 (1996).
5. S. B. Engelsen, JAOCS **74,** 1495 (1997).
6. A. Höskuldsson, Chemom. Intell. Lab. Syst. **23,** 1 (1994).
7. M. Andersson, University of Lund, Sweden, e-mail: Martin.Andersson@teknlk.LTH.se, personal communication.
8. J. H. Holland, *Adaptation in Natural and Artificial Systems* (University of Michigan Press, Ann Harbor, Michigan, 1975).
9. C. H. Spiegelman, M. J. McShane, M. J. Goetz, M. Motamedi, Q. Li Yue, and G. L. Coté, Anal. Chem. **70,** 35 (1998).
10. G. P. McCabe, Technometrics **26,** 137 (1984).
11. W. J. Krzanowski, Appl. Statist. **36,** 22 (1987).
12. H. Martens and T. Næs, *Multivariate Calibration* (Wiley, New York, 1993), 2nd ed.
13. S. Wold, Technometrics **20,** 397 (1978).
14. S. B. Engelsen, E. Mikkelsen, and L. Munck, Progr. Colloid Polym. Sci. **108,** 166 (1998).
15. C. A. Andersson, "Optimization Approaches to Selection of Ranges of Variables in Bi- and Multi-linear Calibration", in preparation for J. Chemom.
